# Establishment of sex-specific liver transcriptomes and H3K9me3 profiles during sexual maturity: the impact of maternal obesity

**DOI:** 10.1186/s13293-025-00767-8

**Published:** 2025-10-21

**Authors:** Ajay K. Yadav, Arianna Harris-Kawano, Romil Saxena, Guanglong Jiang, Jia Ji, Hongyu Gao, Kok Lim Kua, Núria Morral

**Affiliations:** 1 Department of Medical and Molecular Genetics, Indiana University School of Medicine, Indianapolis, IN USA; 2 Department of Medicine, Indiana University School of Medicine, Indianapolis, IN USA; 3 Department of Pathology and Laboratory Medicine, Emory University School of Medicine, Atlanta, GA USA; 4 Center for Medical Genomics, Indiana University School of Medicine, Indianapolis, IN USA; 5 Department of Pediatrics, Indiana University School of Medicine, Indianapolis, IN USA; 6 Department of Anatomy, Cell Biology & Physiology, Indiana University School of Medicine, Indianapolis, IN USA; 7 Center for Diabetes and Metabolic Diseases, Indiana University School of Medicine, Indianapolis, IN USA; 8 Department of Biochemistry and Molecular Biology, Indiana University School of Medicine, Indianapolis, IN USA; 9 Department of Medical and Molecular Genetics, Indiana University School of Medicine, 975 West Walnut Street, room IB 130, Indianapolis, IN 46202 USA

**Keywords:** Cellular differentiation, Liver, Heterochromatin, Histone methylation, Sex dimorphism, Maternal obesity, Western-style diet

## Abstract

**Background:**

The escalating prevalence of metabolic dysfunction-associated steatotic liver disease (MASLD) is closely linked to rising obesity rates. Maternal obesity (MO) is associated with increased susceptibility to metabolic disorders, including MASLD, in the offspring. This elevated risk could be a consequence of epigenetic modifications established during fetal development, a period highly sensitive to the maternal diet. H3K9me3, a hallmark of heterochromatin, plays a vital role in development by silencing gene programs dispensable for differentiated cell types. This study investigated how MO influences gene expression and chromatin architecture in male and female offspring liver, in early postnatal live and upon sexual maturity.

**Methods:**

Female mice were fed a Western-style diet or a control diet before and throughout pregnancy and lactation. The offspring were weaned at 3 weeks and subsequently transitioned to a standard chow diet for 5 weeks.

**Results:**

At 3 weeks, the liver transcriptomes of control offspring were similar between sexes. However, MO disrupted hepatic gene expression in both sexes, leading to the dysregulation of hundreds of genes and alterations in H3K9me3 binding patterns. By 8 weeks, as the mice reached sexual maturity, control offspring showed considerable sex-based gene expression divergence, with over 1,800 genes showing differential expression. These genes were predominantly involved in immune response regulation, cell adhesion and extracellular matrix organization, xenobiotic and glutathione-mediated detoxification, cholesterol metabolism, and lipid partitioning. Furthermore, thousands of differentially bound H3K9me3 peaks were observed between the 3- and 8-week time points. A significant fraction of these peaks were located on the X chromosome in females, suggesting a role in X inactivation. Remarkably, MO offspring displayed incomplete normalization of gene expression, H3K9me3 profiles, and hepatic lipid classes by week 8, underscoring the long-term impact of maternal diet on the genomic and metabolic landscape.

**Conclusions:**

Collectively, this study highlights inherent sex differences in liver gene expression, and suggests that H3K9me3 plays a role in establishing sex-specific liver function during sexual maturation. Moreover, MO disrupts these patterns, which are not fully corrected by 5 weeks of postnatal dietary normalization.

**Supplementary Information:**

The online version contains supplementary material available at 10.1186/s13293-025-00767-8.

## Highlights

• Hepatic gene expression is similar in males and females in early postnatal life (3 weeks).

• Maternal obesity similarly disrupts the liver’s lipid, gene expression, and H3K9me3 profiles in male and female offspring during early postnatal life.

• A 5-week control diet post-weaning fails to fully reverse maternal obesity-induced abnormalities.

• Upon the onset of sexual maturity (8 weeks), >1,800 genes exhibit sex-specific differential expression.

**Supplementary Information:**

The online version contains supplementary material available at 10.1186/s13293-025-00767-8.

## Abstract (plain language summary)

The escalating prevalence of metabolic dysfunction-associated steatotic liver disease (MASLD) is linked to the rising global rates of obesity. Maternal obesity (MO) increases the offspring’s susceptibility to severe metabolic disorders, including MASLD. This increased vulnerability may be due to changes in epigenetic modifications –changes in how genes are “packaged”, which affects their activity (expression). H3K9me3, a critical epigenetic modification, plays a vital role in proper development by silencing non-essential gene programs. Our study investigated how MO influences the gene expression and H3K9me3 patterns in male and female offspring livers. Female mice consumed a Western-style diet or a control chow diet before and throughout pregnancy/lactation. Offspring were weaned at 3 weeks, then transitioned to chow for 5 weeks. At 3 weeks, control offspring liver gene expression was largely similar across sexes. However, MO profoundly disrupted gene expression in both sexes, dysregulating hundreds of genes and significantly altering H3K9me3 patterns. By 8 weeks, upon reaching sexual maturity, control offspring exhibited substantial sex-based divergence in liver gene expression, impacting over 1,800 genes crucial for immune function, lipid metabolism, and detoxification. Notably, at 8 weeks, MO offspring displayed an incomplete recovery. Even after 5 weeks on a healthy diet, their liver gene expression, H3K9me3 profiles, and fat profiles remained partially abnormal. This highlights the impact of a mother’s diet on her offspring’s genomic and metabolic programming, with consequences that are not easily reversed.

**Supplementary Information:**

The online version contains supplementary material available at 10.1186/s13293-025-00767-8.

## Background

Metabolic dysfunction-associated steatotic liver disease (MASLD, previously known as non-alcoholic fatty liver disease) affects 25–30% of adults [1] and is a risk factor for type 2 diabetes and cardiovascular disease [2, 3]. The increasing prevalence of MASLD is primarily driven by rising obesity rates, promoted by sedentary lifestyles and overnutrition. The presence of obesity among women of reproductive age has important implications for fetal health. Maternal obesity is associated with adverse outcomes in the offspring, including higher risk of developing MASLD [4].

The liver is a vital organ that plays a central role in multiple aspects of physiology, including the regulation of carbohydrate and lipid metabolism, protein and bile synthesis, innate immunity, and detoxification. Hormonal signals are key regulators of liver metabolism. Insulin, glucagon, cortisol, growth and thyroid hormones, influence various metabolic pathways to support the liver’s functions and maintain whole body energy homeostasis [5]. In addition, sex hormones have profound effects on liver metabolism. Estrogens enhance insulin sensitivity, reduce *de novo* lipogenesis, and inhibit inflammation, protecting pre-menopausal women against MASLD [6]. Androgens also have strong effects on liver metabolism. Alterations in testosterone levels are associated with MASLD [6]. Thus, the liver exhibits sex hormone-specific responses to nutritional stressors.

Recent studies suggest that exposure to the maternal nutrient milieu during fetal development may influence gene expression via epigenetic alterations that remain after birth, thereby affecting gene expression and risk of metabolic disease [7]. Histone modifications are key regulators of chromatin structure and gene expression that can be influenced by various factors, including diet and hormonal signals [8]. Histone H3 lysine 9 trimethylation (H3K9me3) is a hallmark of heterochromatin, and is written by methyltransferases SUV39H1/H2, SETDB1/2, G9A, and G9A-like protein. H3K9me3 is read by heterochromatin protein 1 (HP1), which recruits other proteins that mediate transcriptional repression [9]. H3K9me3 is found in centromere and telomere repeats, as well as transposable elements (TEs) such as long and short interspersed nuclear elements (LINEs and SINEs, respectively), silencing them and thereby maintaining genomic stability [10]. H3K9me3 has also been associated with euchromatic regions, repressing the expression of genes by binding to their promoters [11, 12]. This histone modification plays a key role in development, regulating tissue-specific gene expression and silencing gene programs not necessary for differentiated cells [9]. Recent studies have revealed dynamic changes in H3K9me3 patterns in response to environmental stimuli. Aberrant H3K9me3 distribution patterns can disrupt normal gene expression programs, contributing to disease initiation and progression. In fact, the dysregulation of H3K9me3 profiles has been linked to cancer [13, 14], neurodevelopmental disorders [15], MASLD [16, 17], and the aging process [18].

Despite well-documented sexual dimorphism in liver function, the mechanistic basis for these sex differences remains incompletely understood. Key outstanding questions include: (1) whether sex-specific transcriptional programs are established during fetal development or emerge postnatally; (2) whether epigenetic remodeling of heterochromatin —specifically through the H3K9me3 histone modification—contributes to sex-specific changes of the liver transcriptome upon sexual maturity; and (3) how maternal obesity differentially influences hepatic gene expression and epigenetic modifications in male and female offspring across developmental stages. To address these gaps, we analyzed sex-specific gene expression and H3K9me3 profiles in both male and female offspring at 3 weeks and 8 weeks of age, and examined the impact of maternal obesity across these same time points. Our data show that in 3-week-old mice, the liver transcriptomes of male and female offspring from control diet-fed dams are nearly identical. However, maternal obesity markedly altered the liver transcriptome of both sexes. Between 3 and 8 weeks of age, gene expression and H3K9me3 profiles underwent substantial changes, affecting cell adhesion, cytoskeleton organization and innate immune response pathways. This study provides novel insight into how maternal obesity shapes the developing liver epigenome and transcriptome and potentially influences long-term metabolic health in the offspring.

## Methods

### Animals

**Fig. 1:**
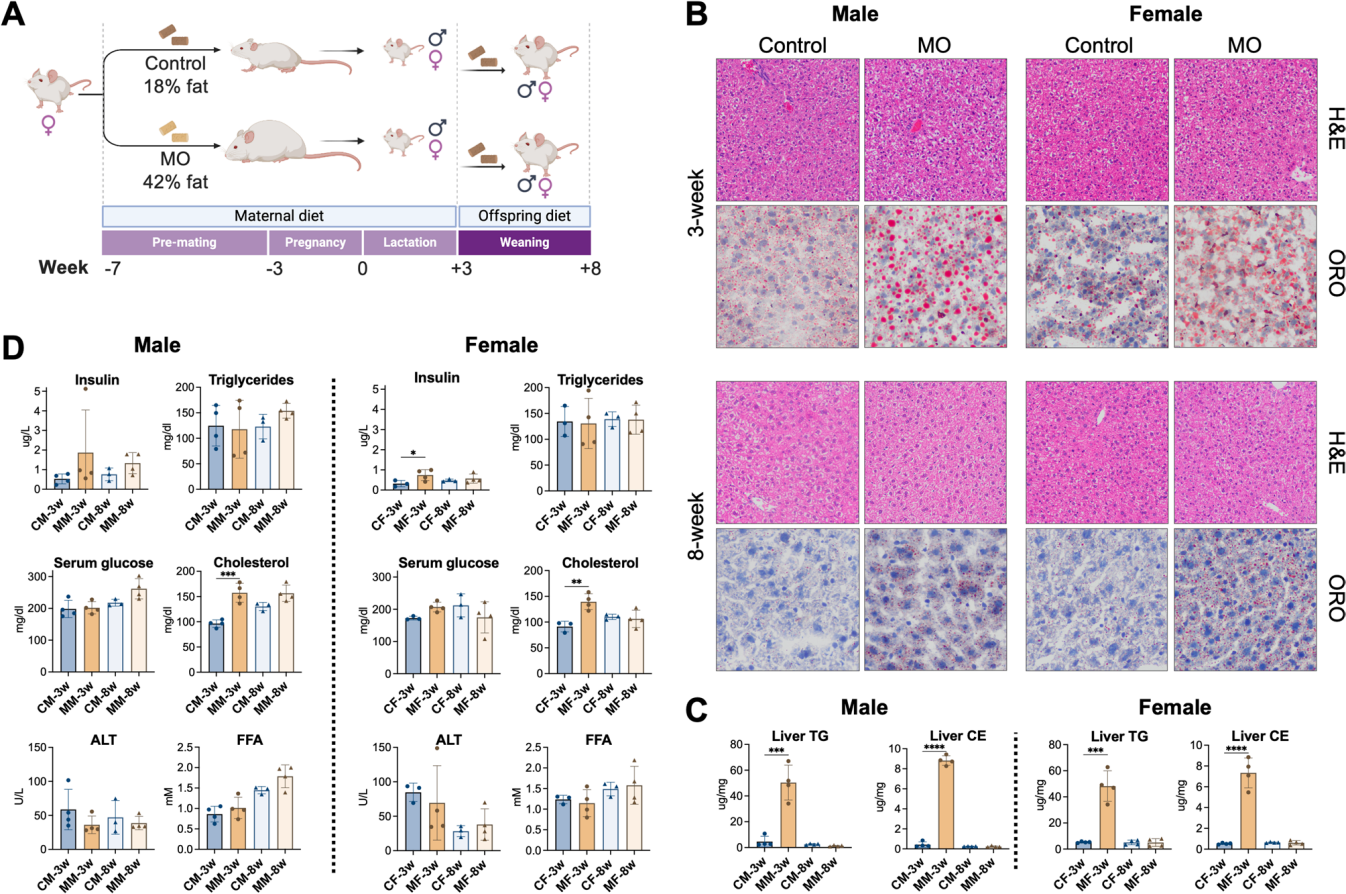
Experimental design and phenotypic characterization. (**A**) Experimental design. Female dams were fed a control or a Western-style diet, as described in the Methods section. Male and female offspring of control or obese dams were analyzed on postnatal day 21 (3 weeks), prior to weaning and the onset of puberty (*n* = 4/group). After weaning, male and female mice received a regular chow diet for 5 weeks (8-week time point; *n* = 4/group). MO, maternal obesity; (**B**) Hematoxylin/eosin and Oil Red O staining of liver sections (*n* = 3–4/group). Hematoxylin/eosin, 100x; Oil Red O, 200x; (**C**) Quantification of liver triglycerides (TG) and cholesterol esters (CE) (*n* = 4/group); (**D**) Serum biochemistries (*n* = 3–4/group). Bars represent standard deviation, *p < 0.05, **p < 0.01, ***p < 0.001

All animal procedures were conducted in accordance with the National Institutes of Health guidelines and were approved by the Indiana University School of Medicine Institutional Animal Care and Use Committee. A standard 12 h light/12 h dark cycle (7 AM/7 PM) was maintained throughout the experiments. Mice were housed in a BSL1 room and had free access to food and water. Experimental animals included littermate control mice derived from an in-house *Reg3g*^fl/fl^ colony (see Supplemental Methods for breeding details). Female dams (Fig. 1A) were fed (i) a Western-style diet (TD.88137, Inotiv, IN: 42 kcal% fat, 15.2% protein, 42.7% carbohydrate; contains 341.46 g/kg sucrose, as well as 1.5 g/kg cholesterol), or (ii) a chow diet (2018SX, Envigo, IN: 18 kcal% fat, 24% protein, 58% carbohydrate), as described [19, 20]. Mice were analyzed on postnatal day 21 (3 weeks), prior to weaning. Additional cohorts were analyzed at 8 weeks of age, after receiving regular chow diet for 5 weeks following weaning [19]. Male and female offspring were included in the studies (*n* = 4/group). Mice were euthanized under fed (*ad libitum*) conditions. The liver was collected and snap-frozen in liquid nitrogen, embedded in optimal cutting temperature (OCT) compound and frozen in liquid nitrogen, or fixed in 10% buffered formalin for histological analysis.

### Tissue histology

Liver tissue was fixed for 24 h in 10% buffered formalin and transferred to 70% ethanol. Specimens were dehydrated through a graded series of ethanols (45 min per step), cleared in two changes of xylenes (45 min each) and infiltrated through four changes of melted paraffin (~ 60 °C; 45 min each). For general histological examination, four-micron (4 μm) sections were cut using a rotary microtome equipped with disposable steel knives, floated onto microscope slides, dried, and stained with hematoxylin and eosin. For lipid visualization, liver tissue snap-frozen in optimal cutting temperature (OCT) compound was sectioned and stained with Oil-Red-O. Processing and staining were performed by the Histology Services Core at Indiana University School of Medicine.

### Serum biochemistries

All serum biochemistries were analyzed by the Center for Diabetes and Metabolic Diseases Translational Core at Indiana University School of Medicine. Insulin was analyzed using a mouse insulin ELISA kit (#10–1247-10, Mercodia, Uppsala, Sweden). Alanine aminotransferase (ALT), glucose, triglycerides, and total cholesterol were measured using a Roche Integra 400 plus analyzer (#20764957322, #04404483190, #20767107322, #03039773190, respectively; Roche Diagnostics, Mannheim, Germany). Free fatty acids (FFA) were measured using a Randox Daytona Clinical Chemistry Analyzer (#FA115; Randox Laboratories, Crumlin, UK).

### Liver tissue lipids

Liver triglycerides and cholesterol ester profiles were analyzed by the Hormone Assay & Analytical Services Core at the National Mouse Metabolic Phenotyping Centers, Vanderbilt University. Briefly, lipids were extracted from ~ 100 mg of tissue using the method of Folch-Lees [21]. Individual lipid classes were separated by thin layer chromatography. Triglycerides and cholesteryl esters were scraped from the plates and methylated using BF_3_/methanol as described [22]. The methylated fatty acids were extracted and analyzed by gas chromatography. Gas chromatographic analyses were carried out on an Agilent 7890 A gas chromatograph equipped with flame ionization detectors and a capillary column (SP2380, 0.25 mm x 30 m, 0.20 μm film, Supelco, Bellefonte, PA). Helium was used as the carrier gas.

### RNAseq

Total RNA was extracted using a RNeasy Midi kit (Qiagen, Valencia, CA, USA) following the manufacturer’s protocol (*n* = 4/group). RNA integrity was evaluated by RNA ScreenTape analysis (Agilent Technologies, Santa Clara, CA, USA). RNA library construction and sequencing were performed by the Center for Medical Genomics at Indiana University School of Medicine. Briefly, mRNA libraries were generated from 100 ng RNA using the KAPA mRNA Hyperprep kit (Roche, Indianapolis, IN, USA) for 3-week time point samples and the Illumina Stranded mRNA Prep, Ligation kit (lllumina) for 8-week time point samples. Paired-end 100-bp reads (3-week) or 150-bp (8-week) were generated using the Illumina NovaSeq 6000 platform (~ 40 million reads/sample). Approximately 85% of reads were uniquely mapped to the mouse genome reference mm10 using Spliced Transcripts Alignment to a Reference (STAR) [23]. To evaluate the quality of the RNAseq data, the number of reads that fell into different annotated regions [exonic, intronic, splicing junction, intergenic, promoter, untranslated region (UTR), etc.] of the reference genes was determined using bamUtils [24]. Low-quality mapped reads (including reads mapped to multiple positions) were excluded, and featureCounts [25] was used to quantify gene expression. The data were filtered using a counts per million (cpm) cutoff with each gene of at least 15 raw reads in at least four of the samples, normalized using TMM (trimmed mean of M values) method, and subjected to differential expression analysis using edgeR [26]. Differentially expressed genes between the groups were analyzed separately for 3-week and 8-week time points. False discovery rate (FDR) was computed from p-values using the Benjamini-Hochberg procedure. For gene clustering, Ingenuity Pathway Analysis (IPA) [27] was used, with statistical significance determined by Fisher’s Exact Test p-value (Benjamini-Hochberg corrected, *p* < 0.05).

### ChIPseq

Chromatin immunoprecipitation was carried out as previously described [8]. Frozen liver tissue (50 to 100 mg) from four biological replicates per group was used to isolate chromatin using the MAGnify Chromatin Immunoprecipitation System kit (Invitrogen, Carlsbad, CA). Tissue was minced in cold D-PBS and chromatin was immediately crosslinked with 1% methanol-free formaldehyde for 5 min. Crosslinking was stopped with glycine to a final concentration of 0.125 M. Tissue was homogenized at 4 °C in 1-mL syringes by passing ~ 20 times through 18G followed by 21G needles. After addition of lysis buffer, cells were sonicated using Bioruptor UCD-300 (30 cycles of 30 s ON, 30 s OFF) to generate DNA fragments of an average size of 100–400 bp. Sonicated chromatin was immunoprecipitated overnight at 4 °C with an antibody specific to H3K9me3 (#17–625, Millipore, Burlington, MA). The shearing quality of DNA was assessed by D5000 ScreenTape Assay (Agilent Technologies Inc., Santa Clara, CA).

Purified ChIP and input DNA samples were used for library preparation using Illumina TruSeq Nano DNA LT Library Prep Kit (Cat# FC-121–4001), including end-repair, dA-tailing, indexed adaptor ligation and amplification. Each resulting indexed library was quantified and its quality accessed by Qubit and Agilent Bioanalyzer. Multiple libraries were pooled in equal molarity. The pooled libraries were then denatured and neutralized, before loading onto an Illumina NovaSeq 6000 for 75-bp paired-end sequencing (Illumina, Inc.). Approximately 20 million reads per library were generated. To identify consensus peaks across different experimental conditions, the raw sequencing reads (fastq files) were aligned to the mouse reference genome (mm10/GRCm38, GENCODE M25) using the Burrows-Wheeler Aligner [28]. Broad peaks were then identified with MACS2 and peaks in ENCODE blacklist regions were subsequently removed. Consensus peaks across different experimental conditions were defined as those present in at least three replicates. Subsequently, the consensus peak-sets from each condition were merged to pinpoint overlapping peaks, treating male and female samples separately. Peaks exceeding 500 bp were divided into bins of 500 bp each. Sequence reads in the consensus peaks were counted using featureCounts (v1.6.2) [25]. Differential binding analysis was conducted with edgeR (v4.3.3) using exact test [26]. The peaks were annotated in terms of genomic features using ChIPseeker [29], including those located in the transcription start site (TSS), exon, 5’ UTR, 3’ UTR, intronic or intergenic. To visualize peaks and the alignment to the mouse genome bigWig files were uploaded onto the IGV genome browser (v2.18.2) [30].

Ingenuity Pathway Analysis [27] was employed to cluster differentially bound (DB) H3K9me3 peaks between the 3-week and 8-week time points. For this analysis, DB peaks with an FDR < 0.05 (3-week versus 8-week comparisons) or p-value < 0.001 (MO versus control diet comparisons) were included. The fold change was not considered for the pathway analysis (i.e., no z-scores were generated). Statistical significance of canonical pathways was determined by Fisher’s Exact Test p-value (Benjamini-Hochberg corrected, *p* < 0.05).

### Statistical analysis and data graphing

For RNAseq and ChIPseq analysis, four biological replicates per group were selected, consistent with previous studies in mouse models, where this number has been shown to provide sufficient statistical power to detect biologically meaningful differences [8, 31]. Data visualization and graphing were prepared using SRplot [32], R, and GraphPad Prism v10.3.1. GraphPad Prism was used to calculate p-values by one-way ANOVA with Šídák’s correction for multiple comparisons. A p-value < 0.05 was considered statistically significant. Data are presented as the arithmetic mean ± standard deviation (SD).

## Results

To investigate the influence of diet on the offspring during embryo development, dams were fed a Western-style or a control diet for 4 weeks prior to mating, throughout gestation, and during the 3 week lactation period (Fig. 1A), as previously described [20]. Male and female mice born of obese dams developed hepatic steatosis compared to those from dams fed a control diet (Fig. 1B). However, no inflammation, necrosis or fibrosis was observed. To evaluate long-term effects, after weaning, a group of offspring from both MO and control diet dams were switched to a chow diet for 5 weeks (8-week time point, Fig. 1A). Interestingly, both male and female offspring of MO dams exhibited slightly higher density of lipid droplets in hepatocytes compared to offspring of dams fed a control diet (Fig. 1B). While liver triglycerides and cholesterol esters (the main neutral lipids), had reverted to normal levels in MO offspring relative to control offspring (Fig. 1C), the fatty acid composition remained altered (Supplemental Fig. 1). This alteration was particularly notable in triglycerides, with enrichment of saturated fatty acids at the expense of polyunsaturated fats, suggesting that 5 weeks of a control diet is insufficient to fully reverse the effects of developmental and early-life exposure to maternal obesity. Furthermore, in females, serum insulin, triglycerides, cholesterol and free fatty acids were indistinguishable between groups at the 8-week time point (Fig. 1D). Males, however, exhibited a tendency towards having higher serum insulin and lipids in MO offspring (serum cholesterol and glucose showed significant differences when the 3-week and 8-week datasets were analyzed separately).

### Maternal obesity (MO) has a large impact on liver transcriptome profiles

**Fig. 2:**
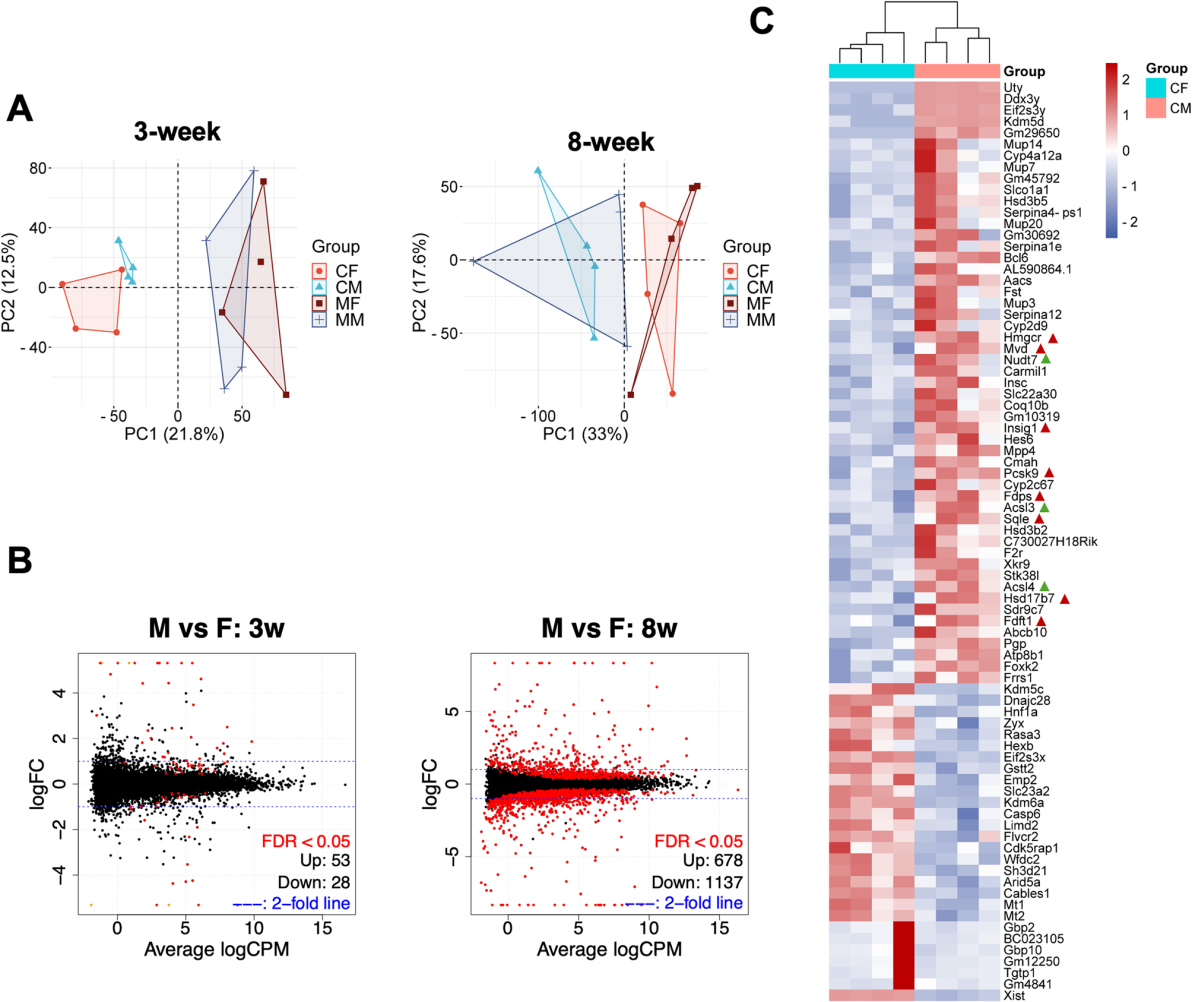
Differences in gene expression between male and female liver at weeks 3 and 8. (**A**) Principal component analysis (PCA) between treatment groups at 3 weeks and 8 weeks; (**B**) Volcano plot of DEGs between males and females at 3 and 8 weeks (*n* = 4/group). Mice were offspring of dams fed a control diet; (**C**) Heatmap of gene expression (log2CPM) differences between control male (CM) and control female (CF) at week 3 (*n* = 4/group). The red triangles highlight cholesterol biosynthesis genes, while the green underscore fatty acid metabolism genes

To determine whether MO affects gene expression, transcriptomic analysis was performed using RNA-seq (mRNA) across all groups. A total of 15,142 genes were found to be expressed in the liver at week 3, and 14,242 at week 8 (Supplemental Tables 1 and 2). Principal component analysis (PCA) was used to examine relationships among groups (Fig. 2A). Remarkably, at 3 weeks, control male and female offspring clustered together, and separately from MO offspring. This suggests that at week 3 the liver gene expression profiles between males and females are similar, and the exposure to MO affects both sexes in a similar manner. Consistently, few genes were found differentially expressed between male and female offspring from control dams at week 3 (81 genes at FDR < 0.05, Fig. 2B). Multiple genes clustered in cholesterol and fatty acid biosynthesis pathways, and were expressed at higher level in males than females (Fig. 2C). Ten of these genes are sex chromosome-encoded, including *Uty*, *Ddx3y*, *Eif2s3y*,* Kdm5d*, and *Gm29650* (Y chromosome), and *Xist*, *Acsl4*, *Kdm5c*, *Eif2s3x*, *Kdm6a* (X chromosome). The estrogen receptor alpha (*Esr1*, major isoform in liver) was not different between males and females at week 3 (Supplemental Fig. 2 A).

**Fig. 3:**
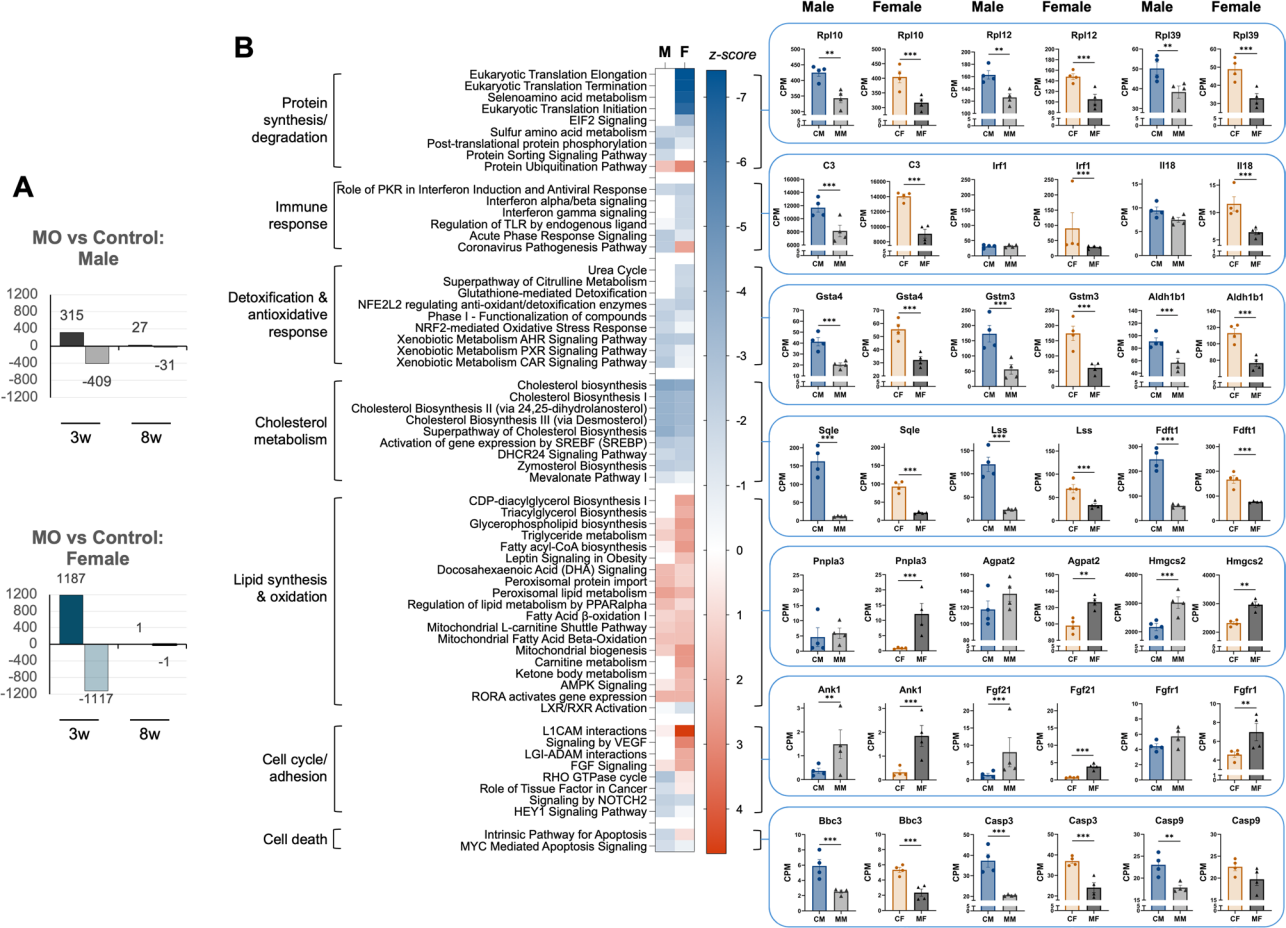
Pathway analysis of DEGs at week 3. (**A**) DEGs between control and MO offspring (*n* = 4/group); (**B**) Heatmap illustrating differences between MO and control diet offspring (males and females). Genes with significant differential expression (FDR < 0.05) between MO and control diet offspring were subjected to pathway analysis. The heatmap displays categories with a Benjamini-Hochberg corrected p-value < 0.05, considered statistically significant. Only categories with z-scores > 1.5 (red, activated) or <−1.5 (blue, inhibited) at least in one of the two sexes, are shown. Examples of genes from different categories are shown (in CPM), along with their corresponding RNAseq p-value. *p < 0.05, **p < 0.01, ***p < 0.001

Relative to control offspring, exposure to MO resulted in hundreds of differentially expressed genes (DEGs) in male and female mice (724 and 2,304, respectively, FDR < 0.05; Fig. 3A). Gene clustering and biological theme enrichment were conducted using Ingenuity Pathway Analysis [27] (Fig. 3B, Supplemental Fig. 2B, Supplemental Table 3). Protein translation/elongation emerged as the most significant clusters in female offspring of MO dams (primarily involving ribosomal genes, *Rpl*, *Rps*), and were downregulated, suggesting a decrease in protein biosynthesis pathways. Although several of these clusters were not significant in MO males, multiple ribosomal genes were similarly downregulated in male offspring (Fig. 3B). Immune-related gene clusters, including ‘Acute phase response signaling’ and ‘Regulation of Toll-Like Receptors (TLR) by endogenous ligand’ (*C3*, *Serpina1*, *Fga/b/d1*), were mostly downregulated in both sexes, with the exception of *Tlr12* and *Tlr5*, which were upregulated 1.7 to 3-fold in both sexes. However, other immune pathways showed sex-specific regulation: ‘interferon alpha/beta signaling’ (*Irf1/3/6/7*, *Isg15*), ’interferon gamma signaling’ (*Gbp2/4/6/7*, *Trim2/35/68*, *Ciita*), were distinctly affected between males and females, with the majority of these genes being unaffected in males and downregulated in females. Remarkably, expression of *Irf2bp2*, was downregulated more than 2-fold in both sexes, consistent with previous reports in mice and humans with MASLD [33].

MO also decreased multiple genes in the cholesterol biosynthesis pathways in both sexes (*Sqle*, *Lss*, *Fdft1*). Instead, fatty acid oxidation (mitochondrial and peroxisomal) and ketogenesis genes were significantly upregulated in males and females (*Hadha/b*, *Decr1*, *Eci2*, *Hmgcs2*). Triacylglycerol biosynthesis emerged as a significantly enriched cluster in females only (*Pnpla3*, *Gpat4*, *Agpat1/2*,* Elovl2/6*, among others). Nevertheless, the expression of key genes implicated in lipid droplet formation, including *Cidec* and *Fitm2*, and the fatty acid transporter *CD36*, were significantly upregulated in both sexes (1.7 to 10-fold). Glutathione-mediated detoxification (*Gsta2/3/4*, *Gstm1/2/3/4*, *Gstp1*) and xenobiotic metabolism (*Nqo1*, *Aldh1b1/3a2/7a1*) pathways were consistently downregulated in both sexes, with a more pronounced decrease in males. Lastly, pathways associated with the cell cycle and cell adhesion molecules (*Ank1/3*, *Cntnap1*, *Alcam*), as well as cell death (*Casp3/9*, *Bbc3*, *Max*), were significantly affected in both males and females.

### Sex dimorphism in the liver transcriptome of sexually mature mice

**Fig. 4:**
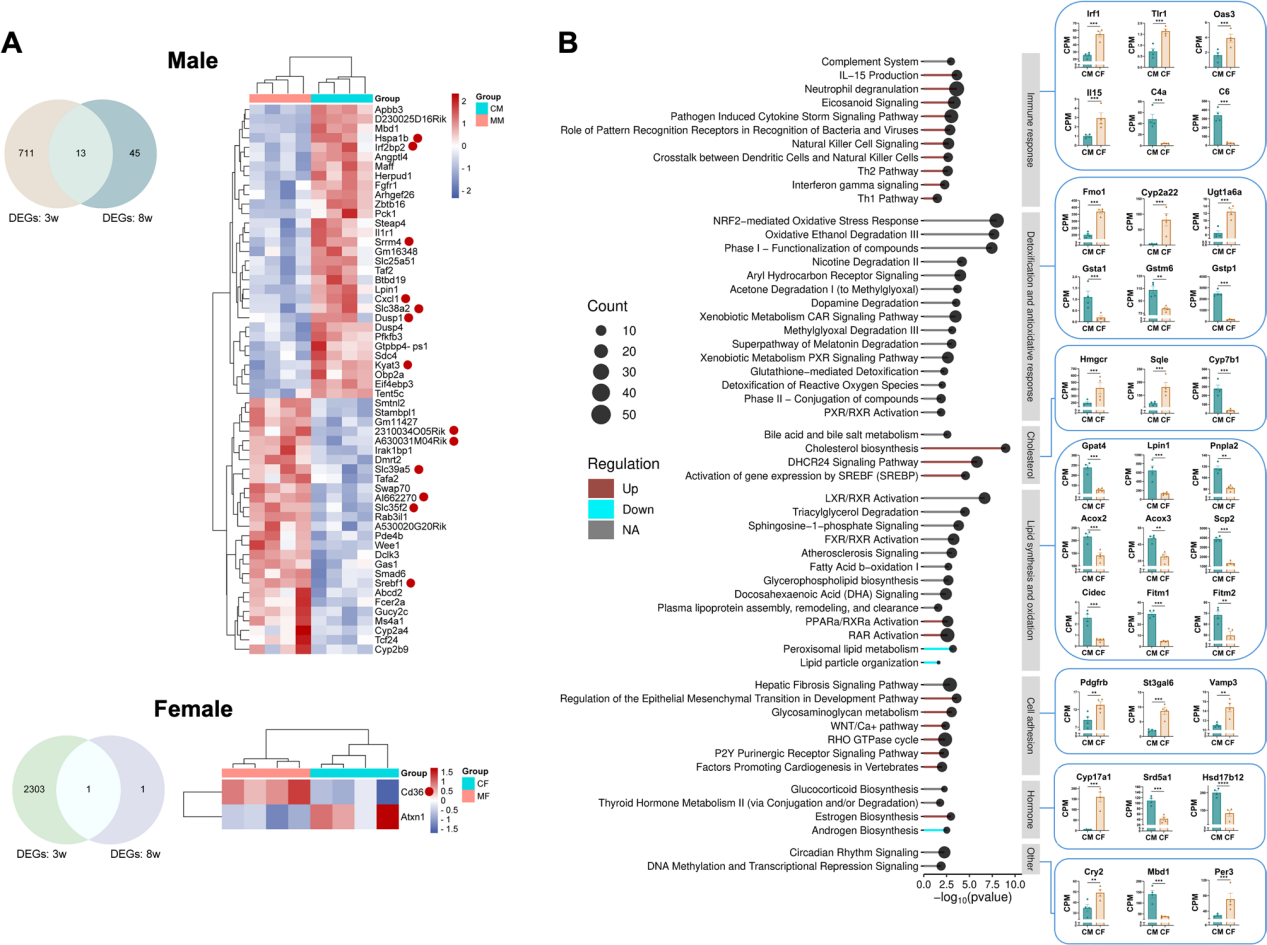
Pathway analysis of DEGs at week 8. (**A**) DEGs between MO and control diet offspring are shown (*n* = 4/group); the Venn diagram highlights genes that are commonly dysregulated at both 3 and 8 weeks; (**B**) Pathway enrichment analysis of genes differentially expressed (FDR < 0.05) between male and female offspring from the control diet group at 8 weeks. Following Benjamini-Hochberg correction, classifications with a p-value < 0.05 were considered significant. Bar colors indicate the z-score: red denotes activation in control females compared to control males, blue denotes inhibition, and gray indicates no directional change (NA; z-scores ≤ 2 or ≥−2). Examples of genes from different categories are shown (in CPM), along with their corresponding RNAseq p-value. *p < 0.05, **p < 0.01, ***p < 0.001

Remarkably, PCA analysis in 8-week-old mice revealed distinct clustering compared to 3-week-old mice (Fig. 2A). Males and females clustered separately, irrespective of MO status, indicating that sex-specific gene expression signatures had already been established in the adult liver. A total of 1,815 genes were differentially expressed (FDR < 0.05) between male and female control offspring (Fig. 2B, and Supplemental Fig. 2 C). Among these was the estrogen receptor alpha (*Esr1*), which was not significantly different at week 3 (Supplemental Fig. 2 A). In contrast to the observations in 3-week-old mice, the number of DEGs between MO and control offspring was markedly lower at 8 weeks: 58 in males and 2 in females (FDR < 0.05, Fig. 4A). In males, only 13 of the dysregulated genes overlapped with those identified at the 3-week time point (Fig. 4A), indicating that changes in gene expression become adjusted as development progresses and diet promotes changes towards normal physiology.

Pathway analysis revealed significant gene expression differences between 8-week-old males and females (Fig. 4B, and Supplemental Table 4). Immune response regulation emerged as the most significantly divergent category. Notable pathways included ‘Interferon γ signaling’ (*Oas3*, *Irf1*, *Trim5*), ‘Eicosanoid signaling’ (*Hpgd*, *Ptgds*), ‘Role of pattern recognition receptors in recognition of bacteria and viruses’ (*C3*, *Ifih1*, *Nod1*, *Tlr1*, *Tlr3*), and ‘Interleukin signaling’ (*Il33*,* Il2rb*,* Il15*). These genes were strongly biased towards being expressed at higher levels in females than males (~ 1.2 to 3-fold). Conversely, several genes that clustered as ‘Complement system’ were expressed at higher levels in males than females (*C2*, *C4a*, *C8a/b*, *C9*; ~1.3 to 13-fold higher).

Other categories that were significantly different between sexes included detoxification-related genes: ‘Nuclear factor erythroid 2-related factor 2 (NRF2)-mediated oxidative stress response’ (multiple *Cyp2/3/4* isoforms, *Sod3*, *Fmo1*), ‘Ethanol degradation’ (multiple *Aldh* and *Cyp2* isoforms), ‘Xenobiotic metabolism Constitutive Androstane Receptor (CAR) signaling pathway’ (multiple *Cyp2/3*, *Sult*, and *Ugt* isoforms), showed a tendency to be more often upregulated in females than males, and some of them (*Sult2a1/2a2/2a3/2a5/3a1/3a2*) were expressed in female liver only (several involved in cholesterol and bile acid secretion). Instead, a few ‘Glutathione-mediated detoxification’ genes (*Gsta1*, *Gstm6*, *Gsr*, *Gstp1/2*; ~1.2 to 13-fold) were lower in females than males, although most enzymes involved in glutathione synthesis (*Gclc*, *Gss*, *Ggt*, *Gpx1/3/4/6/7/8*) showed no significant sex differences. Similarly, a few genes involved in ‘Detoxification of reactive oxygen species’ (*Nox4*,* Prdx5*) were lower in females, while others were expressed at higher levels (*Ncf1*, *Ncf4*, *Cybb*, *Sod3*; ~1.3 to 2-fold).

Notably, genes related to cholesterol metabolism (*Hmgcr*,* Sqle*,* Mvd*,* Idi1*, *Hsd17b7*, *Srebf2*) were expressed at higher level in females (∼1.4 to 2.0-fold), in contrast to what was observed at week 3 (Fig. 2C). Two key bile acid biosynthesis genes were lower in females than males (*Cyp8b1*,* Cyp7b1*; 2.5 to 8.5-fold), although *Akr1d1* was 2-fold higher in females, and the initiating enzymes in the classical and alternative pathways, *Cyp7a1* and *Cyp27a1*, were not significantly different between sexes. In contrast, ‘Peroxisomal lipid metabolism’ genes, such as *Acox2/3*, *Amarc*, enzymes involved in fatty acid oxidation, were slightly higher in males (1.3 to 1.5-fold). Genes implicated in ‘Lipid particle organization’, including *Cidec*, *Fitm1*, *Fitm2*, which play an important role in lipid droplet formation/size, were clearly expressed at higher level in males (4.7, 6.0, and 1.4-fold, respectively). These genes were not significantly different between sexes at week 3, suggesting adult males may be more prone to accumulate large lipid droplets, as seen in previous studies in adult mice fed a high-fat diet [31].

Differences in the expression of genes involved in cell adhesion were apparent between the sexes. Female mice had higher expression of ‘Glycosaminoglycan metabolism’ genes (*Sdc1/3*,* CD44*,* St3Gal6*), ‘Rho GTPase cycle’ (*Dock2/10*, *Pak1*, *Vamp3*), ‘WNT/Ca + pathway’ (*Fzd1/4/7/8*, *Wnt5a*,* Apc2*), ranging from 1.5 to 4-fold higher expression. Multiple genes regulating transcription (*Foxa1/2*, *Mbd1/4*) and circadian rhythm signaling (*Cry2*, *Per2/3*) showed significant sex-based differences in expression. Hormone metabolism pathways showed clear sex-based differences in gene expression. The ‘androgen biosynthesis’ pathway included genes that were expressed at higher levels in males (*Srd5a1*, *Hsd3b2*), while the ‘estrogen biosynthesis’ pathway included genes with higher expression in females (*Esr1*, *Hsd17b7*); other genes displayed significant differences between sexes. For instance, *Cyp17a1*, which is involved in the synthesis of progestins, mineralocorticoids, glucocorticoids, androgens, and estrogens, was expressed 32-fold higher in females than in males. Finally, the category ‘thyroid hormone metabolism II’ (*Dio1*, *Sult1*, *Ugt1*; 1.4 to 2-fold) included genes that were significantly higher in females. Analysis of upstream regulators indicated that estrogen is a key driver of these sex-specific gene expression differences at week 8 (Supplemental Fig. 2D).

### H3K9me3 signatures are associated with changes in liver function

The repressive histone modification H3K9me3 has been linked to the establishment of heterochromatin and cell identity [9]. Thus, we questioned whether this post-translational modification (PTM) has a role in defining sex-specific heterochromatin regions in the liver upon sexual maturity. Comparing H3K9me3 profiles between the 3-week and 8-week time points, a large number of differentially bound (DB) peaks were identified: 6,527 in males (3,583 up and 2,944 down at 8 weeks relative to 3 weeks, FDR < 0.05) and 3,285 in females (1,802 up and 1,483 down at 8 weeks, FDR < 0.05; Supplemental Fig. 3), suggesting that sex hormones influence chromatin structure through this histone PTM.

**Fig. 5:**
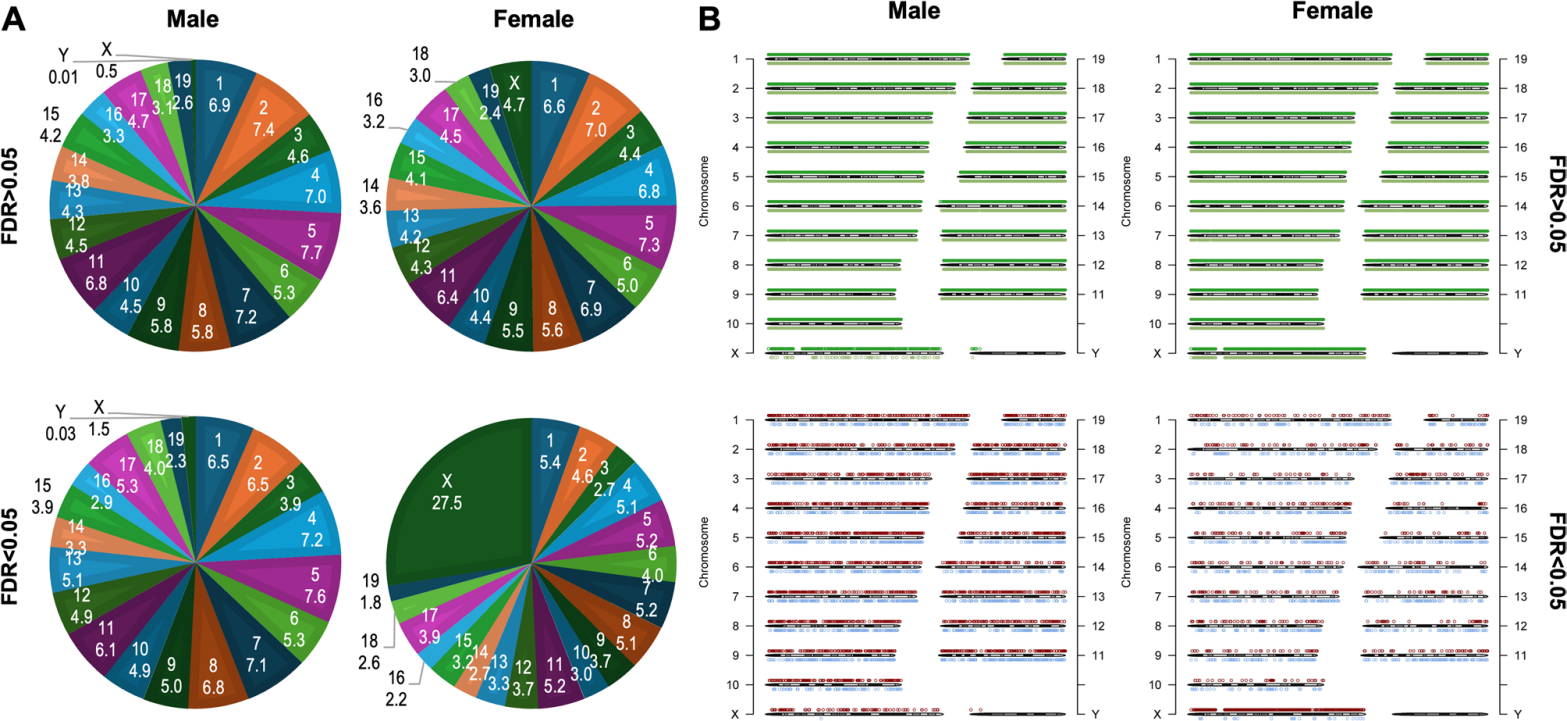
H3K9me3 distribution of peaks in control diet offspring. (**A**) Percentage of H3K9me3 peaks in each chromosome. Numbers indicate the chromosome (top) and the percentage of peaks located within each chromosome (bottom). The distribution of significantly (FDR < 0.05) and non-significantly (FDR > 0.05) DB peaks is shown; (**B**) Distribution of H3K9me3 along each chromosome. Peaks that increased at 8 weeks are shown above each chromosome, while those that decreased are shown below. *N* = 4/group

To map heterochromatic regions across the genome, we analyzed the H3K9me3 distribution in chromosomes. Peaks that did not show significant differences (FDR >0.05) between weeks 3 and 8 were similarly distributed across autosomes in both males and females (Fig. 5). Notably, in females, the X chromosome exhibited proportionally higher representation of H3K9me3 peaks compared to males (4.7% versus 0.5%). Furthermore, approximately 28% of the DB peaks (FDR < 0.05) in females were located on the X chromosome, whereas only 1.5% of DB peaks were found in this chromosome in males (Fig. 5). X chromosome inactivation (XCI) is an important epigenetic process to balance gene dosage between males (XY) and females (XX), with H3K9me3 deposition occurring early in this process [34, 35]. Our data suggest that the lower enrichment of H3K9me3 on the male X chromosome, compared to the female X chromosomes or male autosomes of similar length (e.g., chromosome 2), is likely a consequence of the absence of X inactivation in males.

**Fig. 6:**
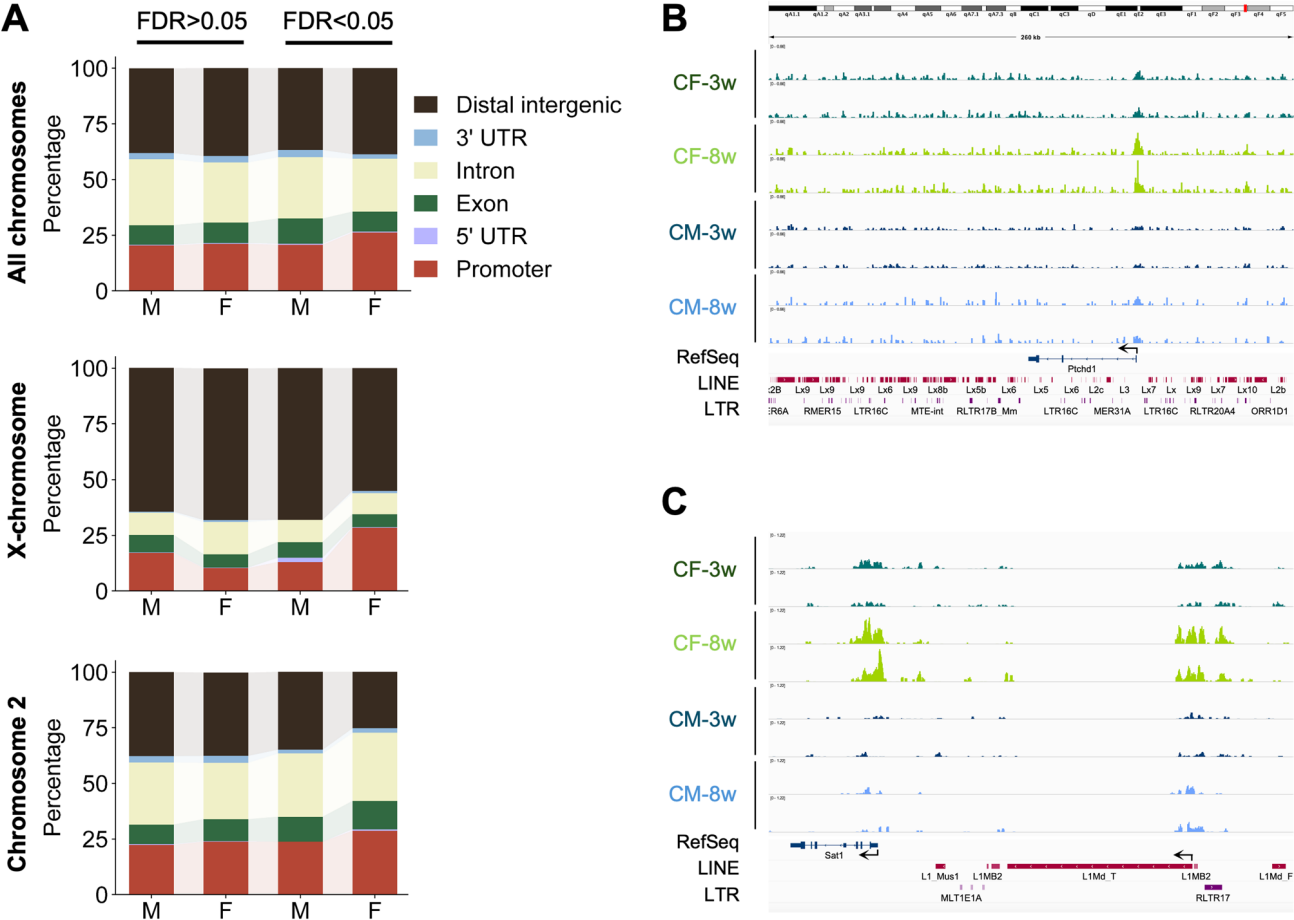
H3K9me3 Distribution in Promoter, Gene Body, and Distal Intergenic Regions. (**A**) H3K9me3 distribution in promoters (± 3 kb from TSS), gene bodies, and intergenic regions, including all chromosomes, or only peaks located on the X chromosome and chromosome 2 (*n* = 4/group). When considering all chromosomes, over 60% of peaks are located within promoters and gene bodies, while approximately 40% are found in intergenic regions. However, this distribution shifts when analyzing the X chromosome alone; (**B**) LINEs are abundant in intergenic regions, particularly in the X chromosome. This is exemplified by a 266 kb region encompassing the *Ptchd1* gene, which is not expressed in liver tissue; (**C**) H3K9me3 enrichment at the promoter region of the *Sat1* gene, as well as in adjacent LINE elements located on the X chromosome

To determine how H3K9me3 contributes to regulate gene silencing, we examined its distribution in promoters, gene bodies and distal intergenic loci. Approximately 20–26% of peaks were located in promoters and 35–43% in gene bodies (Fig. 6A), while 37–38% were found in distal intergenic regions. Remarkably, the distribution on the X chromosome was distinct, with a much larger fraction of peaks found in intergenic regions (55–68%), and a lower representation in gene bodies (16–22%) (Fig. 6A). This pattern likely reflects the higher abundance of LINE-1 (L1) elements on the X chromosome [36], which are enriched in intergenic regions and are known targets of H3K9me3-mediated silencing [9, 37]. Indeed, H3K9me3 peaks were frequently located at the promoter region of L1 elements (Fig. 6B and C).

**Fig. 7:**
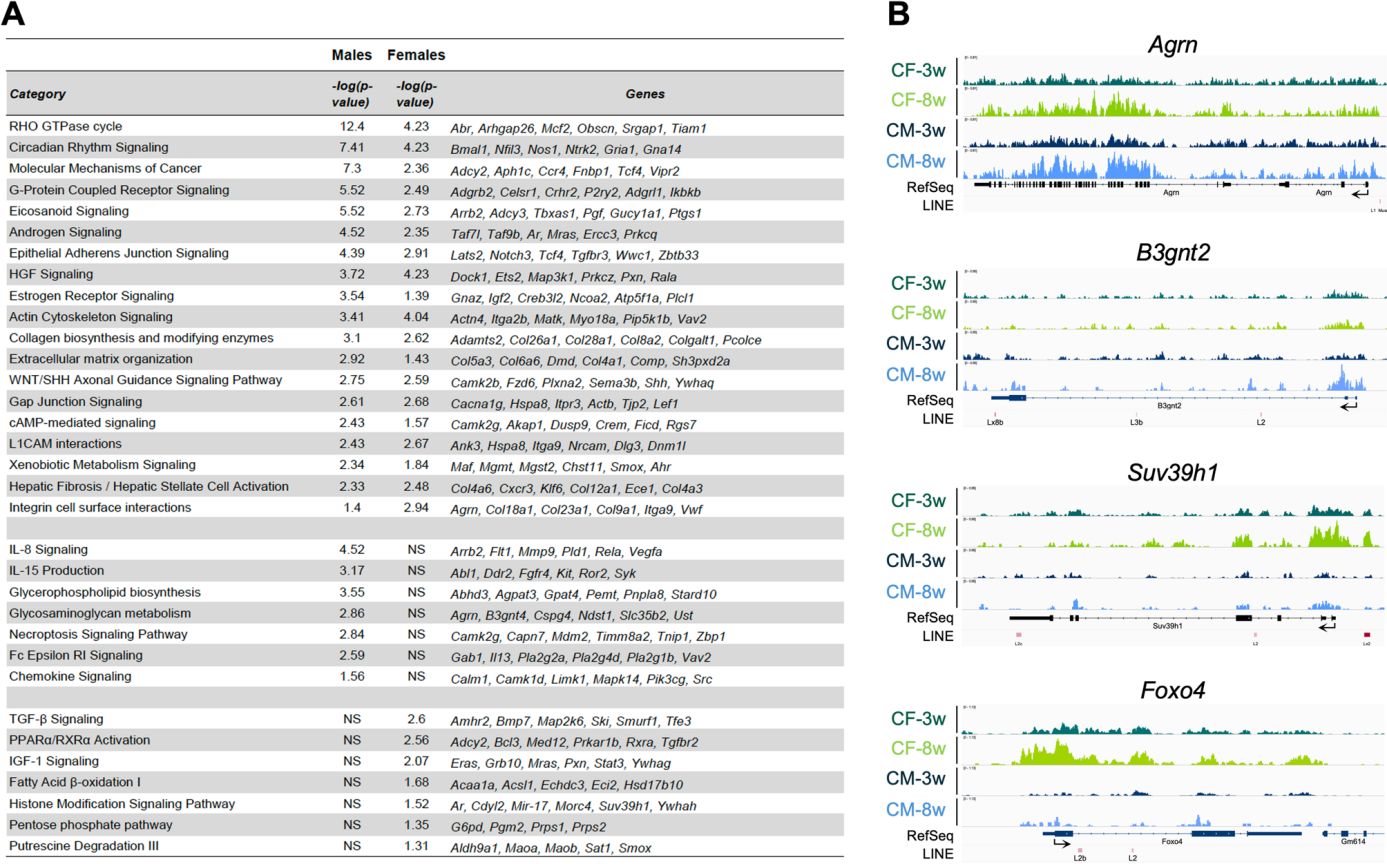
Ingenuity Pathway Analysis of differentially bound (DB) H3K9me3 peaks between 3-week and 8-week time points. DB H3K9me3 peaks (FDR < 0.05) located within promoters and gene bodies were included in the analysis. (**A**) The Table displays significantly enriched Canonical Pathways (Benjamini-Hochberg p-value < 0.05). (**B**) Examples of DB H3K9me3 in promoter or gene bodies are shown

Ingenuity Pathway Analysis was performed on H3K9me3 peaks located within promoter regions and gene bodies. Distal intergenic peaks were excluded from the analysis, as they are predominantly bound to repetitive elements, and their roles in regulating nearby genes is poorly understood. A significant fraction of differentially bound H3K9me3 peaks mapped to genes that clustered within common biological pathways in males and females (Fig. 7, Supplemental Fig. 4, Supplemental Table 5). These pathways included (i) cell adhesion (L1CAM, integrins), cell junction, cytoskeleton organization (actin, Rho GTPase signaling, extracellular matrix), cell proliferation (WNT signaling, Hepatocyte Growth Factor (HGF) signaling), collagen biosynthesis, hepatic fibrosis; (ii) metabolism-related pathways such as cAMP (cyclic Adenosine Monophosphate)-mediated signaling, G-protein coupled receptor signaling, and circadian rhythm signaling; (iii) xenobiotic signaling; (iv) estrogen and androgen signaling. However, several clusters were found to be significantly enriched only in males, including: (i) chemokine signaling, Fc signaling, IL3/8/12/15 signaling, necroptosis signaling; (ii) glycosaminoglycan metabolism and glycerophospholipid biosynthesis. In contrast, other clusters were only significantly enriched in females: (i) histone modification signaling; (ii) Peroxisome Proliferator-Activated Receptor alpha/Retinoid X Receptor alpha (PPARα/RXRα) activation, fatty acid β-oxidation; (iii) Insulin-like growth factor 1 (IGF-1) signaling, pentose phosphate pathway, putrescine degradation. Several female-specific categories included genes located in the X chromosome, and the gene enrichment is likely attributable to H3K9me3 changes associated with X inactivation, rather than inherent sex-based differences. Overall, these data suggest that H3K9me3 plays a role in establishing both tissue- and sex-specific patterns of heterochromatin in the liver upon reaching sexual maturity.

### MO influences H3K9me3 signatures

Feeding a high-fat diet induces reprogramming of chromatin dynamics, leading to changes in gene expression [8, 31]. Thus, we questioned whether the offspring of MO dams would exhibit altered H3K9me3 profiles and whether these aberrant profiles persist after switching to a control diet. Compared to control dams, 3-week-old male and female offspring of MO dams exhibited 443 and 296 DB peaks, respectively (*p* < 0.001, with no peaks found DB at FDR < 0.05), suggesting that diet influences H3K9me3 binding (Supplemental Figs. 5 and 6). The chromosomal distribution of these DB peaks was similar throughout chromosomes (Supplemental Fig. 5B). Most DB peaks were associated with genes involved in transcription regulation, cell differentiation, and cell adhesion (Supplemental Table 6). After five weeks on a chow diet, the number of DB peaks was reduced to 116 in males and 188 in females (Supplemental Figs. 5 and 6), indicating that some aberrant H3K9me3 binding persists, as observed with the gene expression profiles. Remarkably, most DB peaks were found in different loci/genes to those seen at 3 weeks, as observed with gene expression (Fig. 4A). Examination of the top 40 DB peaks (20 upregulated and 20 downregulated) located within promoters or gene bodies, revealed no clear correlation with changes in gene expression: most of the corresponding genes were either not differentially expressed between MO and control animals, or not expressed in liver (Supplemental Table 7). In males, most genes had a very low level of expression (low raw values, Supplemental Table 7). However, in females, most of these marks were in genes expressed at high level (high raw values, Supplemental Table 7).

## Discussion

To advance the development of sex- and age-specific therapeutic treatments, there is growing interest in understanding how sex hormones regulate gene expression and contribute to sexual dimorphism. In this study, we analyzed the liver transcriptome of male and female mice shortly after birth and at sexual maturity. Additionally, we examined whether gene expression in the offspring of obese mothers differs from that in the offspring of dams fed a control diet. Our data shows that male and female 3-week-old offspring of control dams exhibited very similar transcriptome profiles, suggesting minimal sex-specific gene expression at this early stage. However, MO had a strong impact on the liver transcriptome, inducing widespread gene dysregulation, with largely similar transcriptional changes in both sexes, including downregulation of pathways involved in protein synthesis, immune response, detoxification/antioxidant response and cholesterol biosynthesis, along with upregulation of fatty acid oxidation and ketogenesis pathways. Some sex-specific dysregulation was observed, mainly affecting genes involved in the cell cycle, proliferation and cell adhesion.

In contrast, transcriptome profiles at 8 weeks, when sexual maturity was reached, were clearly distinct between males and females. Hundreds of genes were differentially expressed between sexes, affecting predominantly immune response genes, detoxification and antioxidant responses, cholesterol and fatty acid metabolism, cell adhesion, and hormone signaling. Females expressed higher levels of genes involved in various immune-related functions, including interleukins, interferon response genes, and toll-like receptors, consistent with studies in humans showing stronger innate and adaptive immune responses in females [38]. Multiple genes associated with cell adhesion, integrin signaling, and cell migration/proliferation were expressed at higher levels in females compared to males. These include *Grb7*, multiple members of the Frizzled (*Fzd*) and WNT families, as well as genes involved in glycosaminoglycan metabolism. Glycosaminoglycans are polysaccharides that play key roles in cell growth and proliferation, promoting cell adhesion, tissue repair, and coagulation, through interaction with growth factors such as vascular endothelial growth factor (VEGF), fibroblast growth factor (FGF1/2), HGF, and platelet-derived growth factor, among others [39]. Although the precise mechanism through which these sex-based differences influence liver organization is unknown, variations in cell adhesion and extracellular matrix composition influence tissue architecture and/or organization [40, 41]. Furthermore, gene expression differences in glycosaminoglycan genes may have implications for immune regulation, given their role in modulating immunity [42].

Females also showed higher expression of genes associated with liver detoxification processes, particularly Constitutive Androstane Receptor/Pregnane X Receptor (CAR/PXR) xenobiotic metabolism and aryl hydrocarbon receptor signaling, critical pathways that sense toxic byproducts of cellular metabolism and exogenous compounds. These pathways also contribute to the detoxification of oxidized cholesterol and the conversion of cholesterol into bile acids [43].

Additional sex-specific differences involved lipid metabolism. Overall, males showed increased expression of genes implicated in triglyceride synthesis and degradation, as well as lipid particle organization. These included key genes such as *Lpin1*, which mediates the dephosphorylation of phosphatidic acid (PA) to form diacylglycerol (DAG) [44], as well as *Fitm1*, *Fitm2*, and *Cidec*, which participate in the partition of triglyceride towards lipid droplets (*Fitm*) [45] and the transfer of small lipid droplets to larger, more stable droplets (*Cidec*) [46]. These genes were upregulated in adult mice fed a high-fat diet [31]. Notably, increased expression of *Cidec* has also been reported in patients with MASLD [47], and its expression is approximately 10-fold higher in aged male mice (though not in females) compared to younger animals [31]. Taken together, these sex-based differences in gene expression may explain why male mice are more susceptible to developing steatosis and forming large lipid droplets when fed a high-fat diet, while females appear resistant to these metabolic abnormalities [31].

H3K9me3 plays a vital role in maintaining the structure and function of heterochromatin. This histone mark is essential for lineage specification and the maintenance of cellular identity during development, silencing genes not required for a particular cell type [9]. It is well established that hormones regulate transcription and the establishment of sex-specific transcriptional programs via modification of histone PTMs [48]. A number of histone methyltransferase (HMT) and histone demethylase (HDM) enzymes have been identified as regulators of estrogen receptor (ER) signaling [49], including the H3K9me3 demethylase enzyme KDM4B, which is required for appropriate ER target gene expression [50]. Thus, we questioned whether H3K9me3 plays a role in the establishment of sex-specific heterochromatin patterns. Our data show that H3K9me3 profiles undergo significant changes during the transition from early postnatal development to sexual maturity. Changes in H3K9me3 were observed in genes associated with tissue organization pathways, including cell adhesion, cell junction formation, cytoskeletal organization, and collagen synthesis. These pathways also showed significant sex-specific differences in gene expression at the 8-week time point, as discussed above. In addition, changes in H3K9me3 took place in genes involved in metabolism, xenobiotic signaling, circadian rhythm regulation, and hormone signaling. Notably, male-specific pathway enrichment included immune responses and metabolic pathways related to glycosaminoglycan and glycerophospholipid biosynthesis, both of which exhibited sex differences in gene expression at 8 weeks. In contrast, enrichment in females primarily involved metabolism-related pathways, including fatty acid β-oxidation, IGF-1 signaling, the pentose phosphate pathway, and putrescine degradation.

The role of H3K9me3 in silencing TEs and maintaining chromatin stability is well established. However, its function in regulating protein-coding gene expression remains less clear [51]. We observed that approximately 60% of the DB H3K9me3 peaks were located at promoters or within gene bodies, where they may contribute to suppress gene expression by recruiting components of the silencing machinery, or influence expression through other mechanisms. Interestingly, the presence of H3K9me3 within gene bodies has been associated with active transcription in *Drosophila*, and even necessary for appropriate expression [51]. This raises the possibility that a similar regulatory mechanism may exist in mammals, and warrants future research. A recent study examining the effects of maternal obesity (MO) in male offspring demonstrated increased H3K9me3 levels in the liver of MO offspring compared to controls, that persisted even after two weeks on a control diet [52]. The study also reported H3K9me3 enrichment at gene promoters, and in some cases, an association with changes in gene expression [52]. Although our data do not suggest a direct correlation between H3K9me3 and gene expression, it is possible that differential H3K9me3 binding in offspring of MO mice primes the chromatin for aberrant transcription machinery recruitment, altering gene expression under conditions of fasting (mice in this study were *ad libitum* fed). Alternatively, changes in H3K9me3 induced by MO may affect other cellular processes regulated by this modification, including expression of transposable elements, or the DNA damage repair response [53].

In distal intergenic regions, H3K9me3 was predominantly associated with repetitive elements. Transposable elements were historically regarded as parasitic sequences. However, recent studies have shown that they have important biological functions [54], particularly in regulating gene expression during development [10, 37]. For example, H3K9me3-mediated regulation of L1Md (young L1 elements) is essential for maintaining hematopoietic stem cell identity in mice [55]. In addition, H3K9me3 plays a crucial role in X-chromosome inactivation (Xi) by promoting the establishment of heterochromatin [34], and regulating facultative heterochromatin on the Xi [35, 56]. During X inactivation, most genes become transcriptionally repressed, with the exception of a subset known as ‘escapee’ genes, which are not completely silenced and remain partly active —in humans, at >10% of the level seen in the same gene in the active X chromosome [57]. In mice, approximately 3–6% of genes escape X-inactivation, compared to 15–20% in humans [58]. Furthermore, ~ 10–32% of genes exhibit variable X-inactivation status across tissues and/or individuals [59–61]. Escapee genes are important drivers of sexual dimorphism, as several of them encode proteins with broad regulatory effects on autosomes, including histone-modifying enzymes (e.g., *Kdm6a*,* Kdm5c*), helicases (e.g., *Ddx3x*) or eukaryotic translation initiation factors (e.g., *Eif2s3x*) [62]. The mechanism by which X-inactivation spreads along the X-chromosome without affecting escapee genes, is becoming increasingly understood. LINE-1 (L1) elements constitute approximately 20% of the human and mouse autosomes, while accounting for up to 35% of the X chromosome [36, 63]. This enrichment was proposed to function as ‘way stations’ that facilitate the spread of X-inactivation [64]. More recent studies have shown that LINEs promote silencing of the regions of the X-chromosome that become inactive by creating heterochromatic nuclear compartments induced by the initiator of X inactivation, the lncRNA *Xist*. Simultaneously, transcriptionally active young LINEs help repress regions around genes that escape inactivation [65, 66]. Our study suggests that H3K9me3 plays a role in this process, possibly reinforcing sex-specific gene expression patterns established at sexual maturity on X-inactivation.

Lastly, our data indicate that five weeks of a control diet was insufficient to fully reverse the effects of maternal obesity (MO) in offspring. In previous work, we reported increased adiposity at 3- and 8-week time points in male and female offspring of obese dams, despite no significant change in body weight relative to control offspring. At 3 weeks, we observed impaired glucose tolerance in male MO offspring and a trend towards glucose intolerance in females [20]. By week 8, glucose tolerance had normalized in female offspring but remained impaired in males [20]. Likewise, in this study, gene expression improved in both sexes by week 8. However, fewer genes remained dysregulated in females than males, possibly due to estrogen’s protective effects [67]. In line with our findings, recent studies have demonstrated that female offspring of MO dams are protected against MASLD when challenged with a high-fat diet for six months post-weaning, whereas males display steatosis and inflammation [68]. This inflammatory response in males is likely driven by aberrant developmental programming of Kupffer cells (liver macrophages) that persists into adulthood [69].

Consistent with these persistent metabolic and transcriptional changes, MO-induced H3K9me3 alterations at week 3 were not fully resolved by week 8. Furthermore, we observed that MO offspring had a higher density of small lipid droplets, and the fatty acid profiles in triglycerides and cholesterol esters remained altered, even though overall levels of these lipids were undistinguishable between groups. These data suggest that lipid droplet degradation may be a slow process, and that prolonged dietary changes may be required to correct fatty acid profiles. Furthermore, our data raise questions about the impact of elevated saturated and reduced polyunsaturated fatty acid levels on liver physiology, and whether a subsequent Western-style diet challenge in these adult mice would elicit accelerated and/or more severe pathophysiology. Collectively, these results suggest that long-term dietary interventions may be necessary to mitigate the effects of early-life exposure to a Western-style diet.

## Conclusions

**Fig. 8:**
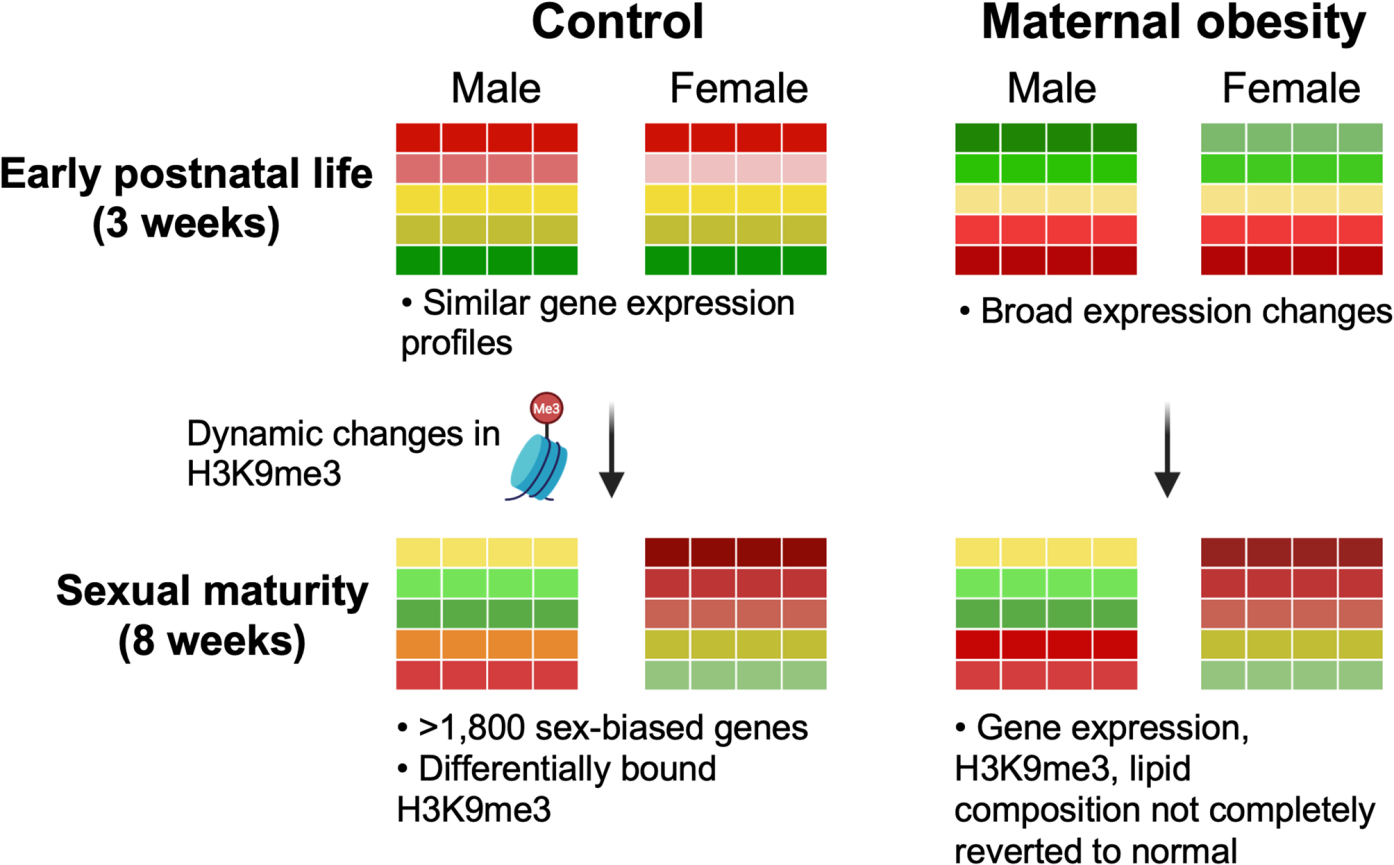
Schematic representation of gene expression changes between time points and diets. Early postnatal liver gene expression is similar between sexes in control offspring. Maternal obesity (MO) disrupts both sexes’ liver transcriptomes and H3K9me3 profiles. Significant sex-specific gene expression divergence occurs by week 8. MO-induced alterations persist despite a 5-week control diet, suggesting lasting impacts on metabolism

In this study we show that the liver transcriptomes of male and female offspring from control diet-fed dams are nearly identical at early post-natal stages (Fig. 8). Maternal obesity markedly altered the liver transcriptome of both sexes in a similar manner. Gene expression and H3K9me3 profiles underwent substantial changes between weeks 3 and 8, affecting cell adhesion, cytoskeleton organization, lipid partitioning, and innate immune response pathways. Importantly, MO-induced alterations were not completely reverted to normal, despite five weeks of postnatal chow diet exposure. This research provides a foundation for understanding the interaction between MO and the establishment of sex-specific H3K9me3 patterns, potentially influencing long-term metabolic health in offspring.

## Supplementary Information

Supplementary Material 1.

Supplementary Material 2.

Supplementary Material 3.

Supplementary Material 4.

Supplementary Material 5.

Supplementary Material 6.

Supplementary Material 7.

Supplementary Material 8.

## Data Availability

ChIPseq and RNAseq files are available from the GEO database (accession numbers GSE304112 and GSE304113).
