# Supplementary material for: Establishment of sex-specific liver transcriptomes and H3K9me3 profiles during sexual maturity: the impact of maternal obesity
Source: Biol Sex Differ. 2025 Oct 21;16:81. doi: 10.1186/s13293-025-00767-8 (PMC12539125; doi:10.1186/s13293-025-00767-8)
Supplement: Supplementary file 8 — Supplementary Material 8. [file 13293_2025_767_MOESM8_ESM.pdf]

Triglycerides

	Male - 3 w			Male - 8 w		
	CM	MM	p-value	CM	MM	p-value
14:0	1.09 ± 0.15	2.16 ± 0.30	ns		0.68 ± 0.79	
16:0	25.75 ± 1.66	29.96 ± 0.19	ns	32.53 ± 2.01	35.14 ± 0.71	0.0016
16:1	2.67 ± 1.06	4.97 ± 0.30	ns	2.57 ± 0.27	2.06 ± 0.41	ns
18:0	3.37 ± 0.96	2.36 ± 0.06	ns	3.09 ± 0.38	4.85 ± 1.25	ns
18:1w9	33.78 ± 7.79	51.29 ± 0.88	<0.0001	29.10 ± 1.14	31.38 ± 1.81	0.009
18:1w7	3.10 ± 1.15	7.66 ± 0.50	ns	2.05 ± 0.17	2.56 ± 0.68	ns
18:2	24.61 ± 8.45	1.60 ± 0.23	<0.0001	28.16 ± 1.63	22.96 ± 1.72	<0.0001
18:3 w3	1.27 ± 0.51	ND		0.57 ± 0.66	ND	
20:3w6	0.21 ± 0.42	ND		ND	ND	
20:4	2.62 ± 1.04	ND		1.33 ± 0.89	0.37 ± 0.74	ns
22:5w3	0.08 ± 0.15	ND		ND	ND	
22:6	1.46 ± 0.68	ND		0.61 ± 0.70	ND	

	Female - 3 w			Female - 8 w		
	CF	MF	p-value	CF	MF	p-value
14:0	0.96 ± 0.10	2.26 ± 0.29	0.001	0.51 ± 0.35	0.62 ± 0.53	ns
16:0	23.85 ± 0.59	29.72 ± 0.67	<0.0001	25.77 ± 1.39	31.58 ± 2.57	<0.0001
16:1	2.16 ± 0.19	5.28 ± 0.82	<0.0001	1.78 ± 0.50	2.11 ± 0.65	ns
18:0	2.53 ± 0.32	2.28 ± 0.22	ns	3.26 ± 0.47	3.48 ± 0.71	ns
18:1w9	30.49 ± 0.49	51.64 ± 1.20	<0.0001	32.88 ± 1.34	33.62 ± 2.31	ns
18:1w7	2.52 ± 0.11	6.97 ± 0.79	<0.0001	2.30 ± 0.26	2.39 ± 0.63	ns
18:2	30.12 ± 0.44	1.86 ± 0.42	<0.0001	28.73 ± 2.21	23.52 ± 5.03	<0.0001
18:3 w3	1.39 ± 0.09	ND		1.06 ± 0.18	0.70 ± 0.48	ns
20:3w6	1.25 ± 0.05	ND		0.50 ± 0.60	0.18 ± 0.36	ns
20:4	2.43 ± 0.19	ND		1.79 ± 0.28	1.23 ± 0.21	ns
22:5w3	0.51 ± 0.34	ND		ND	ND	
22:6	1.79 ± 0.24	ND		1.42 ± 0.21	0.41 ± 0.52	ns

Cholesterol esters

	Male - 3 w			Male - 8 w		
	CM	MM	p-value	CM	MM	p-value
14:0	ND	1.10 ± 0.09		ND	0.88 ± 1.76	
16:0	20.64 ± 3.08	19.62 ± 0.96	ns	27.21 ± 2.39	23.46 ± 1.39	0.0102
16:1	6.72 ± 3.10	13.74 ± 0.68	0.0082	5.20 ± 0.52	5.76 ± 1.53	ns
18:0	9.00 ± 2.92	3.85 ± 0.17	ns	10.98 ± 1.54	11.07 ± 1.74	ns
18:1w9	40.92 ± 7.62	55.73 ± 1.53	<0.0001	30.97 ± 0.89	33.46 ± 3.42	ns
18:1w7	2.37 ± 0.93	5.06 ± 0.36	ns	2.11 ± 0.31	2.06 ± 0.52	ns
18:2	18.47 ± 5.66	0.90 ± 0.10	<0.0001	23.53 ± 2.29	23.31 ± 1.18	ns
20:4	1.88 ± 2.60	ND				

	Female - 3 w			Female - 8 w		
	CF	MF	p-value	CF	MF	p-value
14:0	ND	1.18 ± 0.13		ND	1.05 ± 0.74	
16:0	19.33 ± 2.80	20.02 ± 0.84	ns	18.33 ± 2.10	17.27 ± 2.28	ns
16:1	6.99 ± 0.89	14.76 ± 1.81	<0.0001	5.38 ± 1.19	6.23 ± 1.22	ns
18:0	6.92 ± 2.04	3.62 ± 0.32	0.0306	6.04 ± 0.75	6.33 ± 1.21	ns
18:1w9	42.07 ± 3.22	54.88 ± 2.04	<0.0001	41.53 ± 1.39	45.30 ± 3.65	0.0406
18:1w7	1.78 ± 0.18	4.49 ± 0.63	ns	1.65 ± 0.23	2.11 ± 0.60	ns
18:2	21.31 ± 1.51	1.05 ± 0.19	<0.0001	21.79 ± 2.17	19.62 ± 3.41	ns
20:4	1.60 ± 1.88	ND		4.91 ± 0.41	2.07 ± 2.40	ns

Supplemental Figure 1. Fatty acid classes in liver triglycerides and cholesterol esters. Statistical analysis was conducted using two-way ANOVA with correction for multiple comparisons. The 3-week and 8-week time points were analyzed separately. ND, not detected; CM, control male; MM, maternal obesity male; CF, control female; MF, maternal obesity female.

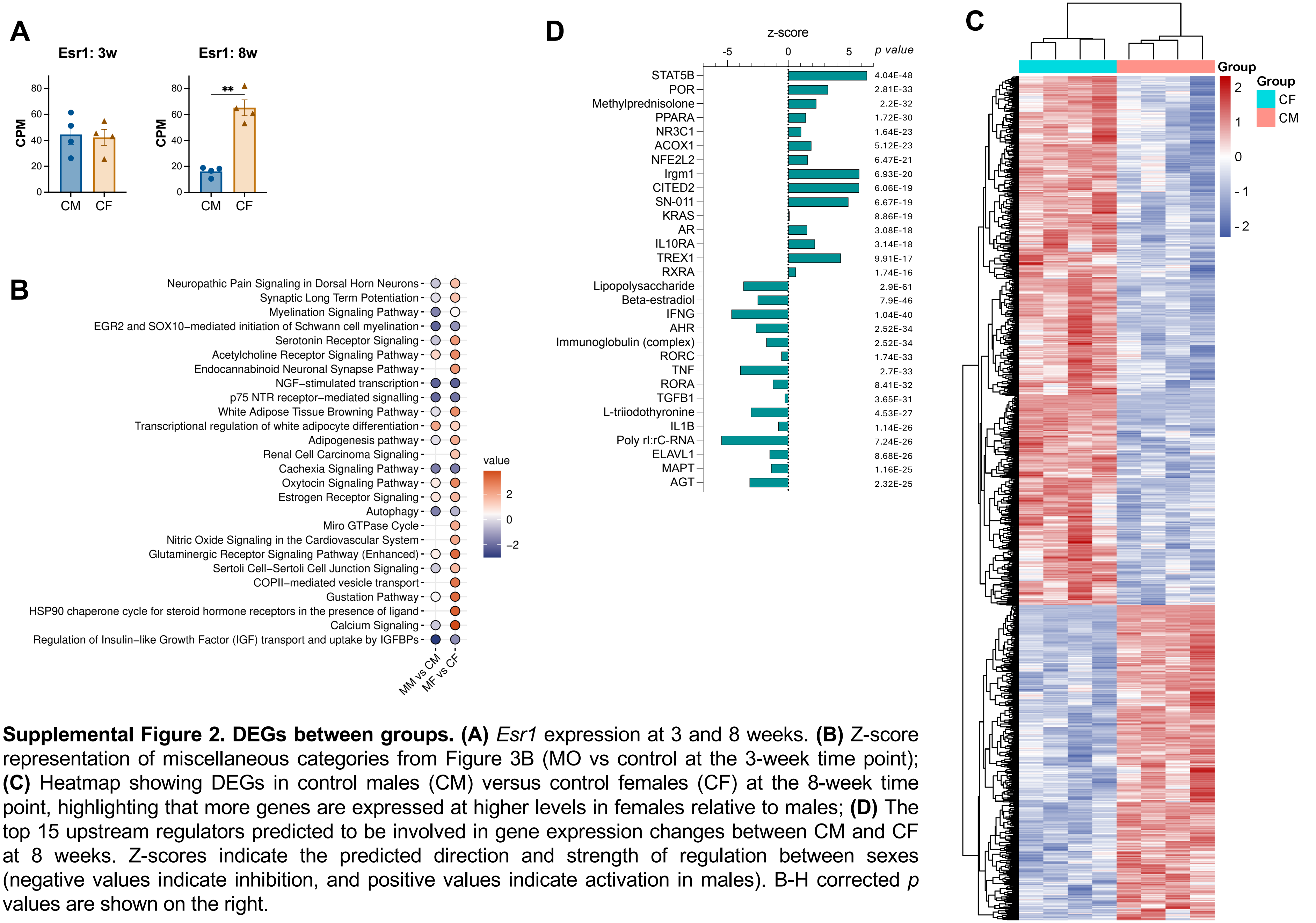

Supplemental Figure 2. DEGs between groups. (A) *Esr1* expression at 3 and 8 weeks. **(B)** Z-score representation of miscellaneous categories from Figure 3B (MO vs control at the 3-week time point); **(C)** Heatmap showing DEGs in control males (CM) versus control females (CF) at the 8-week time point, highlighting that more genes are expressed at higher levels in females relative to males; **(D)** The top 15 upstream regulators predicted to be involved in gene expression changes between CM and CF at 8 weeks. Z-scores indicate the predicted direction and strength of regulation between sexes (negative values indicate inhibition, and positive values indicate activation in males). B-H corrected *p* values are shown on the right.

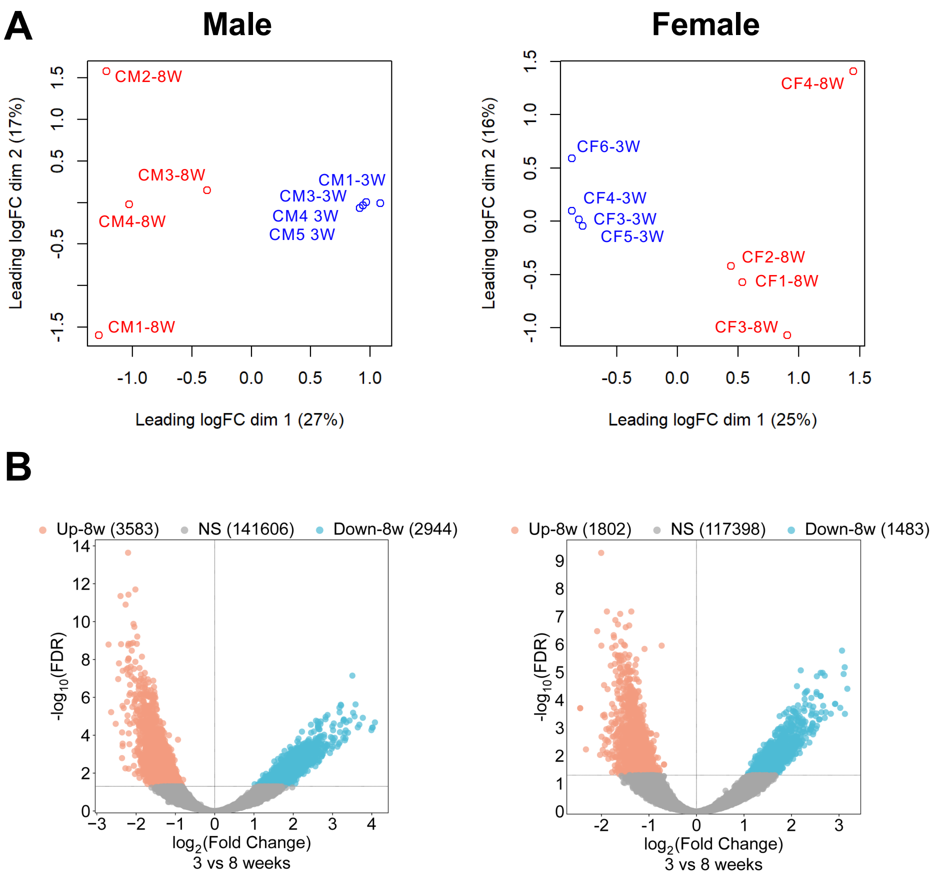

Supplemental Figure 3. Differential H3K9me3 binding (DB) between 3 and 8 weeks. (A) Multidimensional scaling (MDS) plot showing clustering of replicates, with distinct separation especially in the 3-week group. **(B)** Volcano plot depicting differential H3K9me3 binding at 3-weeks relative to 8-weeks, with a significance threshold of FDR<0.05.

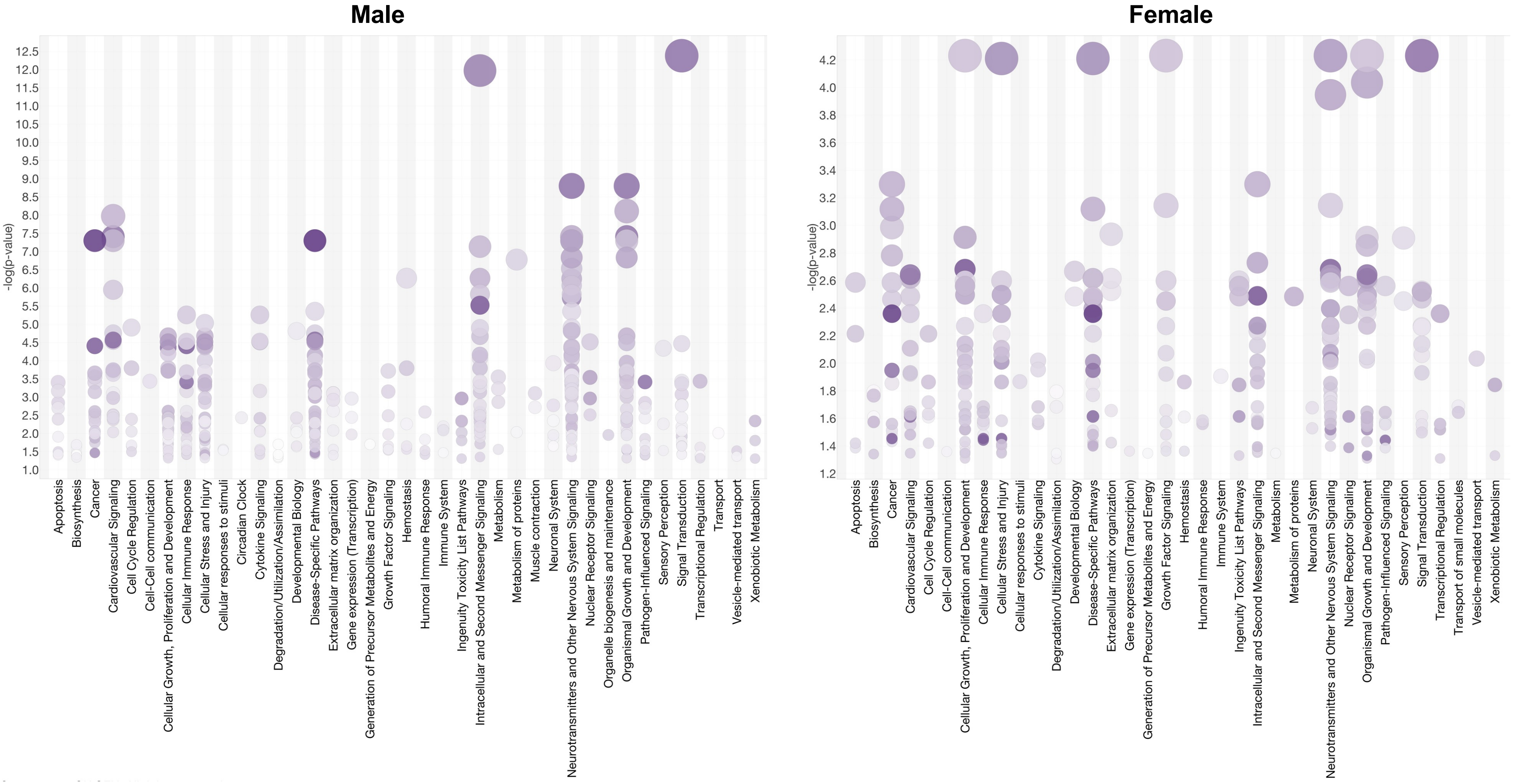

Supplemental Figure 4. Ingenuity Pathway Analysis of differentially bound (DB) H3K9me3 peaks between 3-week and 8-week time points. DB H3K9me3 peaks (FDR<0.05) located at promoters and gene bodies were used in the analysis. The bubble chart displays significantly enriched Canonical Pathway Categories (Fisher's test with B-H correction, p<0.05).

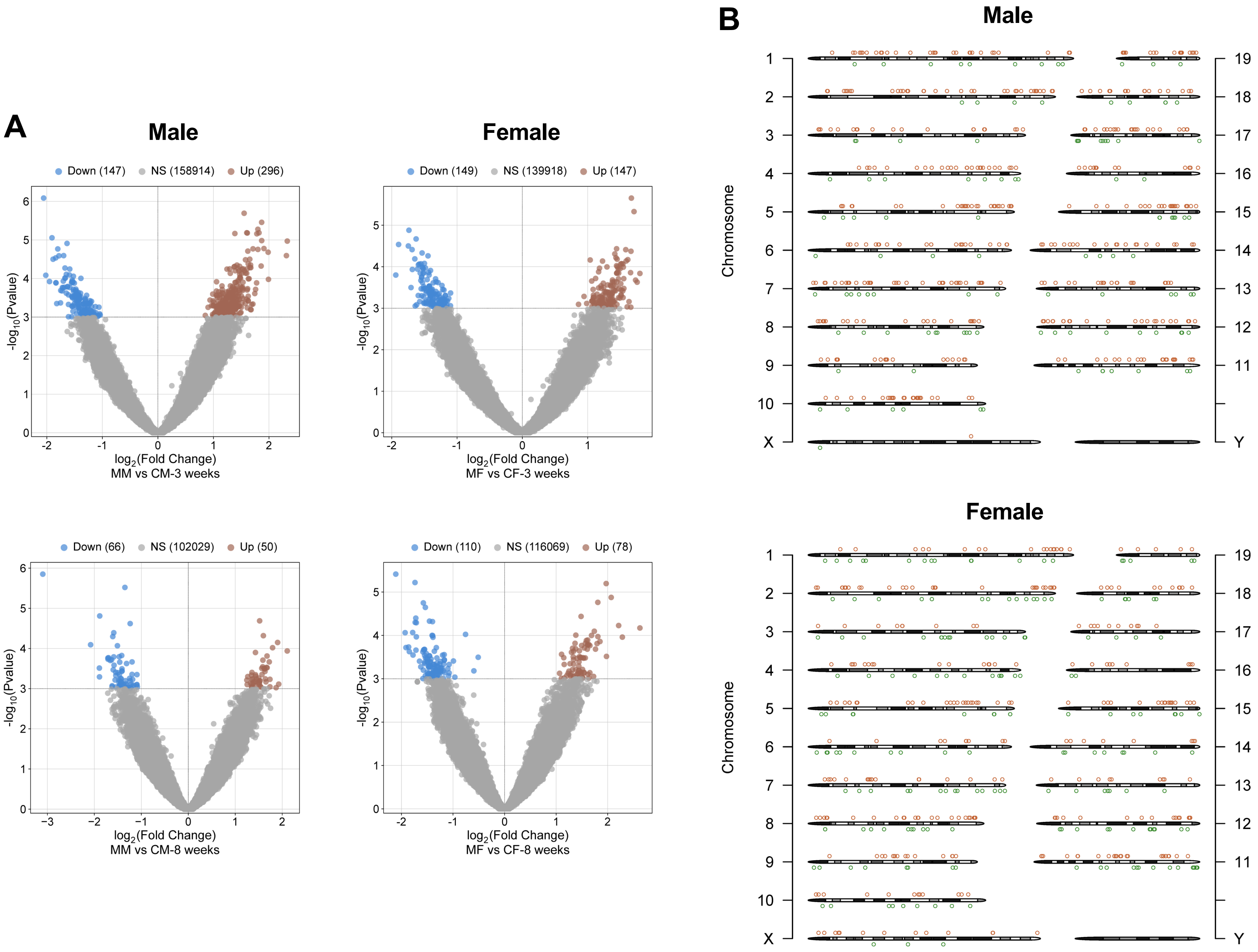

Supplemental Figure 5. DB H3K9me3 peaks in MO relative to control diet (p<0.001). (A) Volcano plot of DB peaks, p<0.001; **(B)** Chromosomal distribution of DB H3K9me3 peaks; Red: DB peaks at week 3; Green: DB peaks at week 8. Females had more changes in H3K9me3 binding on the X chromosome than males.

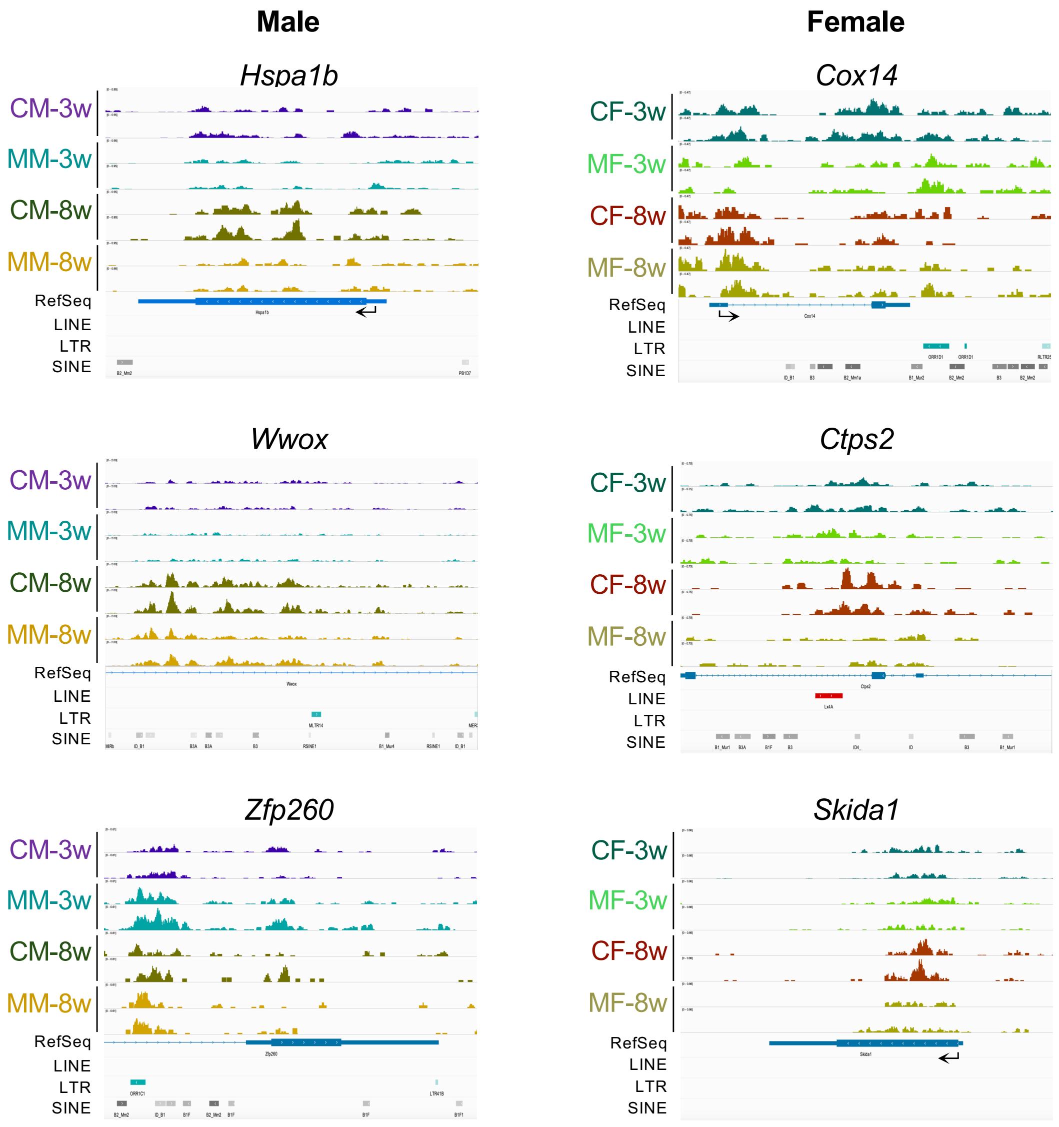

Supplemental Figure 6. H3K9me3 DB between maternal obesity and control diet offspring. Examples show differential deposition of H3K9me3 in promoter (*Skida1*), promoter and gene body (*Hspa1b* and *Cox14*), intron (*Zfp260*, *Wwox* and *Ctps2*).

Establishment of sex-specific liver transcriptomes and H3K9me3 profiles during sexual maturity: the impact of maternal obesity

Ajay K. Yadav et al.

Supplemental Methods

Animal breeding. Mice were housed in a BSL1 room and had free access to food and water. Experimental animals included *floxed* littermate controls derived from an in-house *Reg3g*$^{fl/fl}$ colony maintained on a C57BL/6J background. At 5-6 weeks old, female dams (Fig. 1A) were randomized to receive either (i) a Western-style diet diet (TD.88137, Inotiv, IN), or (ii) a standard chow diet (2018SX, Envigo, IN), as described [19, 20]. After four weeks on the assigned diet, dams were mated with healthy wild-type C57BL/6J males (purchased from The Jackson Laboratory, Bar Harbor, ME). Dams remained on their respective diet throughout pregnancy and lactation. At birth, litters were culled to maximum of 6 pups per dam. Male and female offspring (*Reg3g*-fl/+ or *Reg3g*-fl/fl) were analyzed on postnatal day 21 (3 weeks), prior to weaning and onset of puberty. Additional cohorts were analyzed at the 8 weeks of age after receiving regular chow diet for five weeks following weaning.
